# Therapy-Induced Evolution of Human Lung Cancer Revealed by Single-Cell RNA Sequencing

**DOI:** 10.1016/j.cell.2020.07.017

**Published:** 2020-09-03

**Authors:** Ashley Maynard, Caroline E. McCoach, Julia K. Rotow, Lincoln Harris, Franziska Haderk, D. Lucas Kerr, Elizabeth A. Yu, Erin L. Schenk, Weilun Tan, Alexander Zee, Michelle Tan, Philippe Gui, Tasha Lea, Wei Wu, Anatoly Urisman, Kirk Jones, Rene Sit, Pallav K. Kolli, Eric Seeley, Yaron Gesthalter, Daniel D. Le, Kevin A. Yamauchi, David M. Naeger, Sourav Bandyopadhyay, Khyati Shah, Lauren Cech, Nicholas J. Thomas, Anshal Gupta, Mayra Gonzalez, Hien Do, Lisa Tan, Bianca Bacaltos, Rafael Gomez-Sjoberg, Matthew Gubens, Thierry Jahan, Johannes R. Kratz, David Jablons, Norma Neff, Robert C. Doebele, Jonathan Weissman, Collin M. Blakely, Spyros Darmanis, Trever G. Bivona

**Affiliations:** 1 Chan Zuckerberg Biohub, San Francisco, CA 94158, USA; 2 Department of Medicine, University of California, San Francisco, San Francisco, CA 94158, USA; 3 Helen Diller Family Comprehensive Cancer Center, University of California, San Francisco, San Francisco, CA 94158, USA; 4 Lowe Center for Thoracic Oncology, Dana-Farber Cancer Institute, Boston, MA 02215, USA; 5 Department of Cellular and Molecular Pharmacology, University of California, San Francisco, San Francisco, CA 94158, USA; 6 Department of Medicine, University of Colorado Anschutz Medical Campus, Aurora, CO 80045, USA; 7 Department of Biomolecular Engineering, University of California, Santa Cruz, Santa Cruz, CA 95064, USA; 8 Department of Pathology University of California, San Francisco, San Francisco, CA 94143, USA; 9 Department of Radiology and Biomedical Imaging, University of California, San Francisco, San Francisco, CA 94143 USA; 10 Denver Health Medical Center, Denver, CO 80204, USA; 11 Department of Radiology, University of Colorado, Aurora, CO 80045, USA; 12 Department of Bioengineering and Therapeutic Sciences, University of California, San Francisco, San Francisco, CA 94143, USA; 13 Department of Surgery, University of California, San Francisco, CA 94143, USA; 14 Howard Hughes Medical Institute, University of California, San Francisco, CA 94143, USA

**Keywords:** single-cell RNA sequencing, lung cancer, EGFR, ALK, targeted therapy

## Abstract

Lung cancer, the leading cause of cancer mortality, exhibits heterogeneity that enables adaptability, limits therapeutic success, and remains incompletely understood. Single-cell RNA sequencing (scRNA-seq) of metastatic lung cancer was performed using 49 clinical biopsies obtained from 30 patients before and during targeted therapy. Over 20,000 cancer and tumor microenvironment (TME) single-cell profiles exposed a rich and dynamic tumor ecosystem. scRNA-seq of cancer cells illuminated targetable oncogenes beyond those detected clinically. Cancer cells surviving therapy as residual disease (RD) expressed an alveolar-regenerative cell signature suggesting a therapy-induced primitive cell-state transition, whereas those present at on-therapy progressive disease (PD) upregulated kynurenine, plasminogen, and gap-junction pathways. Active T-lymphocytes and decreased macrophages were present at RD and immunosuppressive cell states characterized PD. Biological features revealed by scRNA-seq were biomarkers of clinical outcomes in independent cohorts. This study highlights how therapy-induced adaptation of the multi-cellular ecosystem of metastatic cancer shapes clinical outcomes.

## Highlights

• scRNA-seq is feasible in metastatic human NSCLCs and reveals a rich tumor ecosystem
• Individual tumors and cancer cells exhibit substantial molecular diversity
• Cancer and tumor microenvironment cells exhibit marked therapy-induced plasticity
• scRNA-seq of metastatic NSCLCs unveils new opportunities to improve clinical outcomes

## Graphical Abstract

**Figure:**
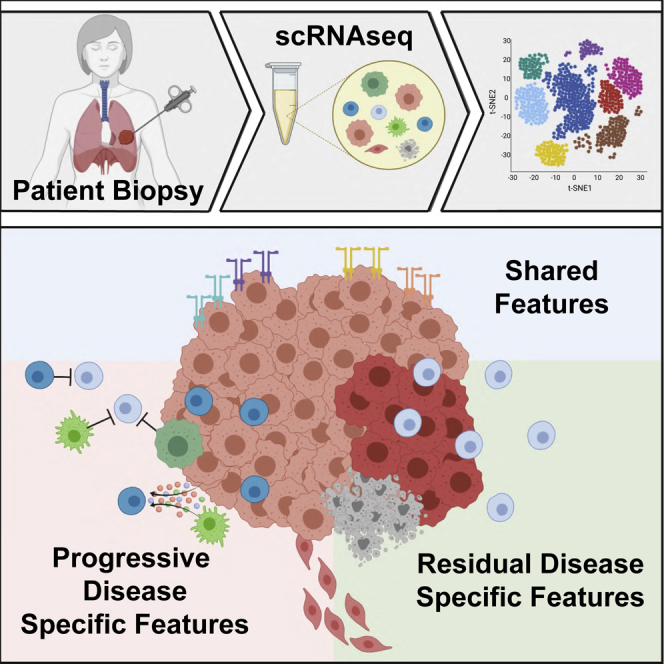

## Teaser

Analysis of metastatic lung cancer biopsies before and after targeted therapy reveals molecular and immune adaptations that shape clinical outcomes.

## Introduction

Heterogeneity is a property of many biological systems and diseases such as cancer. Biological plasticity in cancer cells is one form of heterogeneity that allows for early adaptation to treatment and limits the success of precision approaches for cancer treatment (Xue et al., 2017; Yuan et al., 2019). In addition to cancer-cell intrinsic heterogeneity, cells within the tumor microenvironment (TME) further contribute to tumor heterogeneity in a cancer cell extrinsic manner. While these tumor compartments and tumor heterogeneity have been characterized in many cancer subtypes (Alexandrov et al., 2013; Brannon et al., 2014; Gerlinger et al., 2012; Hata et al., 2016; Lawrence et al., 2013; Lee et al., 2014; Vignot et al., 2015), our understanding of how these properties evolve and interact longitudinally in response to systemic treatment remains incomplete, particularly in metastatic tumors.

Many oncogene-driven cancers such as those with alterations in *EGFR*, *ALK*, *ROS1*, and *BRAF* are treated with targeted therapies against the cognate oncoprotein. This has led to improvements in the clinical outcomes of metastatic solid cancers such as lung cancer and melanoma as well as hematologic malignancies (Flaherty et al., 2012; Mok et al., 2009; Shaw et al., 2013). However, tumors typically respond incompletely and then regrow after acquiring drug resistance. Bulk tumor sampling after progression on targeted therapy has identified resistance mechanisms and demonstrated that tumors become increasingly molecularly heterogeneous following treatment (Blakely et al., 2017; Camidge et al., 2014; McCoach et al., 2018; Rotow and Bivona, 2017).

Single-cell RNA sequencing (scRNA-seq) is one approach to dissect the heterogeneity of complex biological systems (Chung et al., 2017; Darmanis et al., 2017; Tirosh et al., 2016). There is currently a paucity of single-cell studies that sample metastatic malignancies and prior scRNA-seq studies of metastatic disease largely focused on single treatment time points (Chung et al., 2017; Darmanis et al., 2017; Lambrechts et al., 2018; Patel et al., 2014; Tirosh et al., 2016; Wang et al., 2019; Zhang et al., 2019). This is due, in part, to challenges associated with obtaining high-quality samples of metastatic human tumors, particularly at multiple treatment time points.

By developing a custom pipeline, we performed scRNA-seq analyses on advanced-stage NSCLC samples that were obtained from patients before initiating systemic targeted therapy (TKI naive [TN]), at the residual disease (RD) state, which includes samples taken at any time during treatment with targeted therapy while the tumor was regressing or stable by clinical imaging (RD), and upon subsequent progressive disease as determined by clinical imaging, at which point the tumors showed acquired drug resistance (progression [PD]).

## Results

### scRNA-seq Analysis of Advanced-Stage NSCLCs during Targeted Therapy

**Figure 1:**
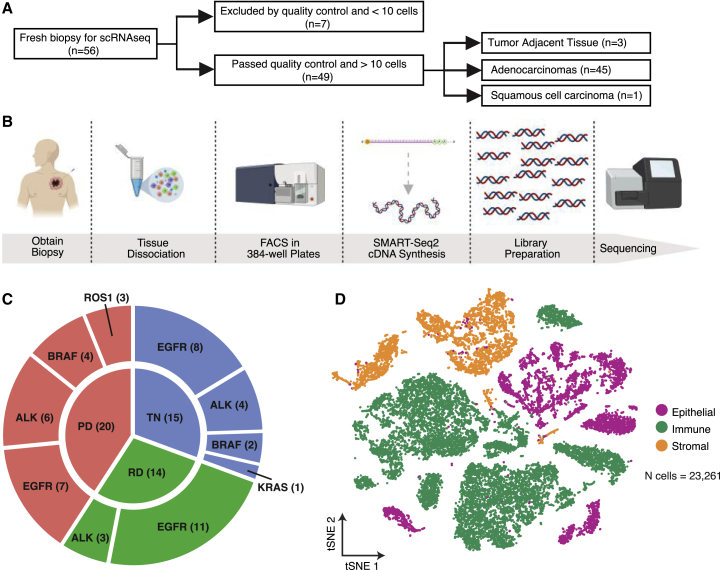
Patient Characteristics and Experimental Overview (A) Consort diagram. 56 biopsies were processed, 49 samples passed quality control. (B) Tissue processing pipeline for scRNA-seq. Patient samples were disaggregated into single cells and sorted into microtiter plates using FACS. cDNA synthesis was performed using the Smart-seq2 protocol, and libraries were sequenced on Illumina platforms. (C) Circle plot of the clinically identified oncogenic driver (outer circle) and treatment time point (inner circle) for each sample. (D) t-stochastic neighbor embedding (t-SNE) plot of all cells colored by their cellular identity (epithelial cells [n = 5,581], immune cells [n = 13,431], stromal cells [n = 4,249]). See also Figure S1 and Tables S1 and S2.

**Figure S1:**
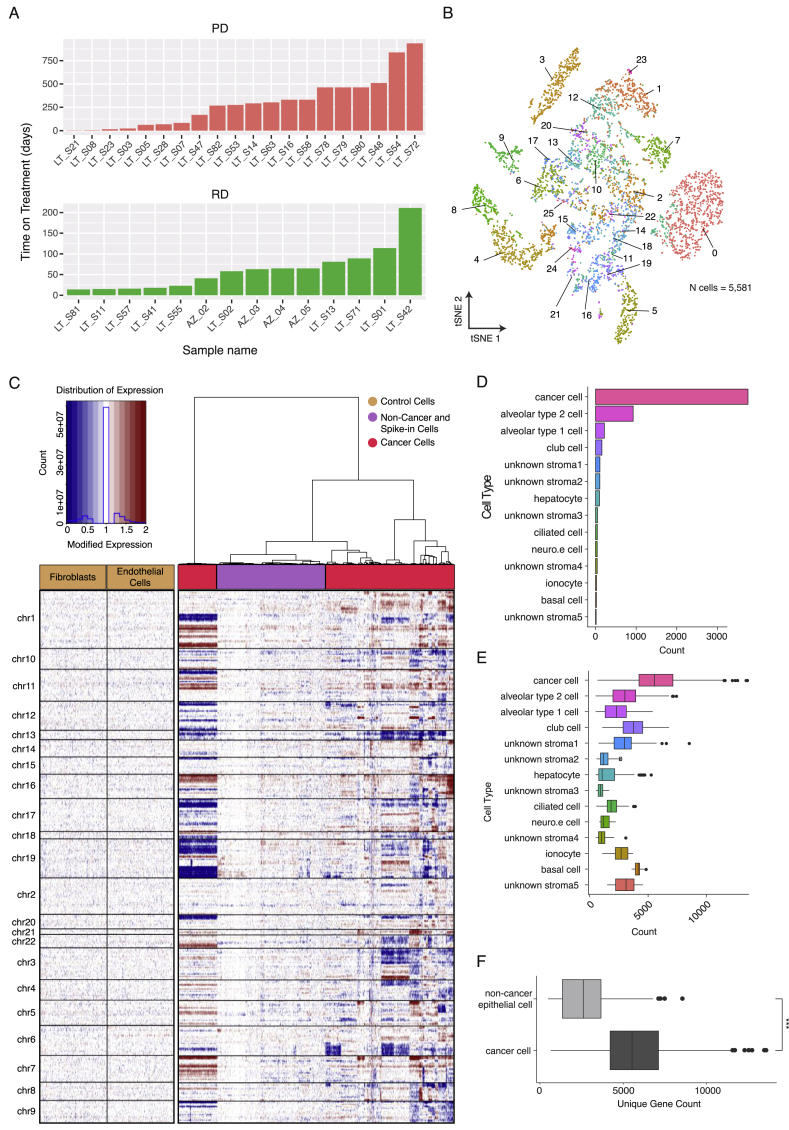
Related to Figure 1 (A) Bar plots of the time interval between treatment start and tissue acquisition for PD and RD tumor samples (B) t-SNE of all epithelial cells (n = 5,581), numbers correspond to individual clusters. (C) Inferred large-scale copy number variations (CNVs) help identify cancer (pink) and non-cancer cells (purple). Epithelial and spike in control cells are included in the x axis and chromosomal regions on the y axis. Amplifications (red) or deletions (blue) were inferred by averaging expression over 100-gene stretches on the respective chromosomes. (D) Bar plot of cell counts for annotated epithelial cells. (E) Bar plot of the number of unique genes across all annotated epithelial cell types. (F) Bar plot of unique gene count of cancer versus non-cancer epithelial cells.

We used scRNA-seq to profile 49 samples (45 lung adenocarcinomas, 1 squamous cell carcinoma, and 3 tumor adjacent tissues [TATs]) (Figure 1A), corresponding to 30 individual patients. We used a customized workflow to isolate viable single cells primarily from small tissue samples as well as surgical resections (Figure 1B). Samples were categorized into three separate time points (TN, RD, or PD) and further subcategorized by oncogenic driver (Figure 1C). Collection time for RD samples is illustrated in Figure S1A. Additional sample details and patient demographics are included in Table S1.

Gene-expression profiles of 23,261 cells were retained after quality control filtering. Following gene-expression normalization, we performed principal-component analysis (PCA) and clustered cells using graph-based clustering on the informative PCA space (n = 20). The resulting cell clusters were annotated as immune, stromal (fibroblasts, endothelial cells, and melanocytes), or epithelial cells (Figure 1D) by established marker genes (Lambrechts et al., 2018; Schiller et al., 2019; Tabula Muris et al., 2018; Treutlein et al., 2014) (Table S2). Epithelial cells (n = 5,581) were subsetted and re-clustered into 26 discrete epithelial clusters (Figure S1B). The number of cells for each cell type and the analyses that each sample was utilized for are detailed in Table S1.

### Clustering-Based Copy-Number Variation Resolves Cancer from Non-cancer Epithelial Cells

Given the association between cancer and large-scale chromosomal alterations, we utilized copy-number variation (CNV) from RNA expression to classify epithelial cells as either cancer or non-cancer (Patel et al., 2014; Puram et al., 2017; Tickle et al., 2019; Tirosh et al., 2016; Venteicher et al., 2017); compared to fibroblasts and endothelial cells (controls), cancer cells displayed larger changes from relative expression intensities across the genome (Figure S1C). Three TAT samples were included in this analysis and the majority of cells originating from these samples were classified as non-cancerous (Table S1). We compared the average CNV score of samples among treatment time points (TN, RD, PD) and found it to be consistent. The non-cancer epithelial cell clusters (n = 16) were further annotated into cell subtypes (Figures S1D and S1E).

As noted by others (Zhang et al., 1997), we found that cancer cells expressed an elevated number of unique genes compared to non-cancer cells (Figure S1F). The difference in the number of uniquely expressed genes was not explained by sequencing depth (Pearson correlation = 0.19).

Cancer cells were identified in 44 of the original 49 tumor biopsy samples including a small fraction of cells originating from each of the TAT samples (0.57%–1.8% of total TAT obtained cells). Given that TAT cells may represent an intermediate cellular state between normal and cancer cells, their presence at low frequency is unsurprising and has been described previously (Aran et al., 2017; Kadara et al., 2014).

**Figure S2:**
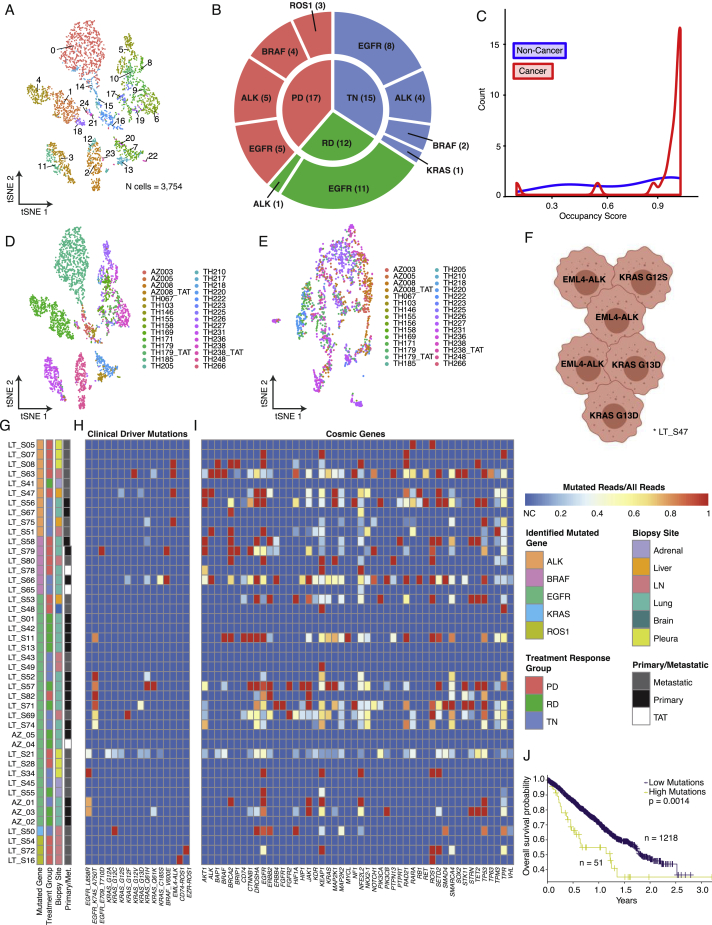
Related to Figure 2 (A) t-SNE plot of 3,754 cancer cells from 44 samples, numbers indicate individual clusters. (B) Circle plot illustrating the clinically identified oncogenic driver (outer circle) and time points (inner circle) of each biopsy, only for cancer cells. C) Density distribution of cluster occupancy of cancer (red) and non-cancer (blue) epithelial cell clusters, calculated as the percentage of the highest contributing individual patient over the total number of cells for that cluster. (D) tSNE of cancer cells colored by patient. (E) tSNE of non-cancer epithelial cells colored by patient. (F) Illustration of heterogeneity of primary driver mutated cancer cells found in exemplary sample LTS47. (G) Clinical characteristics of the 44 NSCLC samples in which at least one cancer cell was identified. Columns indicate clinically identified mutated gene, treatment response time point (TN, RD, PD), biopsy site, and primary or metastatic sample origin, respectively. (H) Cancer cell mutational landscape for each patient sample as determined by scRNaseq represented as a heatmap. Color indicates the number of mutant reads for each genomic region and sample divided by the total number of reads for that region in that sample, NC:No Coverage over the specific genomic region. (I) Mutational landscape of COSMIC tier 1 genes. Color indicates the number of mutant reads for each genomic region and sample divided by the total number of reads for that region in that sample, NC:No Coverage over the specific genomic region. (J) Kaplan-Meier plot showing overall survival of 1269 NSCLC patients within the MSK-Impact dataset. Patients were stratified by high (> = 2) and low (< 2) mutations from the 141 mutations that are present in both the MSK-Impact dataset and panel (I).

All cancer cells (n = 3,754) were re-clustered, resulting in 25 unique clusters (Figures S2A and S2B). For each of the 25 clusters, we calculated the number of cells of the highest contributing individual patient over the total number of cells for that cluster for both non-cancer and cancer epithelial cells (patient occupancy) (Figures S2C–S2E). The majority of cancer cell clusters were patient specific, having high patient occupancy scores, similar to prior reports (Chung et al., 2017; Darmanis et al., 2017; Jerby-Arnon et al., 2018; Neftel et al., 2019; Puram et al., 2017; Tirosh et al., 2016). Conversely, non-cancer cell types exhibited lower patient occupancy (Figure S2E). Thus, patient-specific malignant cell clustering reflected the unique molecular signatures of an individual patient’s tumor rather than technical artifact.

### scRNA-seq Analysis Reveals a Rich Complexity of Expressed Gene Alterations in Cancer Cells

**Figure 2:**
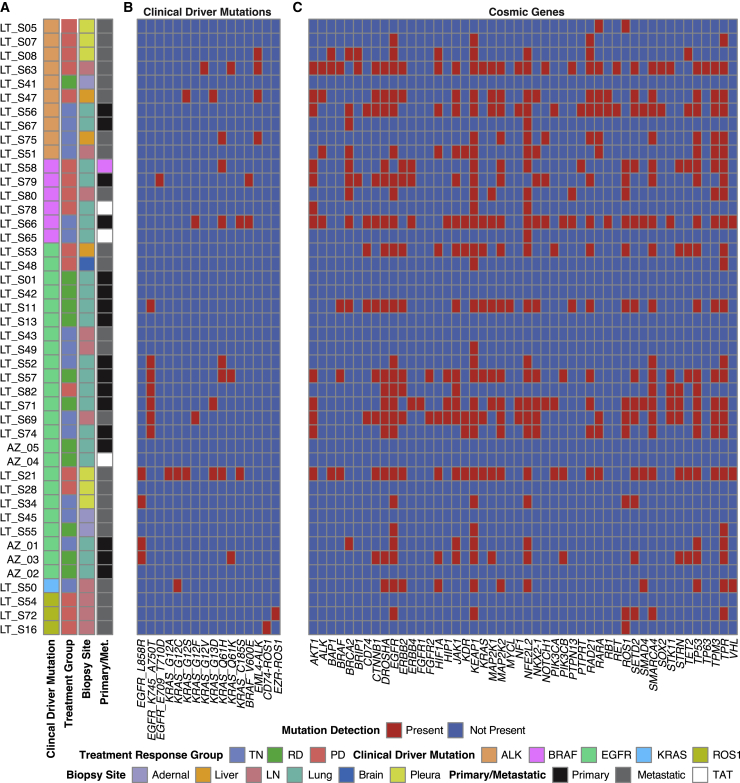
scRNA-seq Infers Patient Mutational Status and Reveals a Complex Mutational Landscape in Cancer Cells (A) Clinical characteristics of the 44 NSCLC samples in which at least one cancer cell was identified. Columns indicate clinically identified mutated gene, treatment response time point (TN, RD, PD), biopsy site, and primary or metastatic sample origin, respectively. (B and C) Cancer cell mutational landscape for each patient sample as determined by scRNA-seq represented as a binarized heatmap across driver genes (B) and COMSIC tier 1 genes (C). Red indicates the presence of mutation while blue indicates that no mutation was identified. See also Figure S2 and Tables S2 and S3.

Additional genetic alterations can co-exist with a primary targetable oncogenic “driver” alteration (e.g., oncogenic *EGFR*, *ALK*, *KRAS*) and may help promote tumor progression and limit therapy response (Blakely et al., 2017; Kim et al., 2019; Scheffler et al., 2019; Yang et al., 2019). We queried scRNA-seq transcripts from each cancer cell to identify somatic alterations (Figures 2A–2C; Figures S2G–S2I). In the 44 of 49 biopsy samples that contained cancer cells, we identified 20 samples harboring the clinically known oncogenic driver (Figure 2B; Table S3). This percentage is consistent with the potential drop-out occurrence in scRNA-seq analyses (Kharchenko et al., 2014). In 24 samples where we did not identify the clinically known oncogenic driver, no cells expressed the gene of interest, thus not allowing mutation detection for that gene (Figure S2H; Table S3). In 11 of the 20 samples (55%) where we identified the clinically actionable oncogene, we also identified an additional oncogenic alteration that was not detected in clinical-grade bulk nucleic acid testing of tumor from the same patient (i.e., occult genetic alterations) **(**Figure 2B; Figure S2H). An example is sample LTS47. This tumor was determined to harbor an *EML4-ALK* oncogenic gene rearrangement by clinical-grade bulk DNA analysis. scRNA-seq additionally revealed that this sample contained cancer cells harboring *KRAS* G13D and *KRAS* G12C occult mutations (Figure S2F). Given the potential of dropout in scRNA-seq data, we cannot conclude whether the *ALK* fusion and the *KRAS* mutations co-exist within the same cell. However, neither population of *KRAS* mutant cells showed evidence of the *ALK* gene rearrangement. This sample was obtained from the patient after multiple lines of therapy, which could have allowed for evolution of multiple mechanisms of resistance (Doebele et al., 2012; Hrustanovic and Bivona, 2015; Shaw and Engelman, 2013). Loss of an oncogenic driver is also a mechanism of resistance (Lovly et al., 2017; Tabara et al., 2012; Xu et al., 2018a), although given the limitation of scRNA-seq we were not able to determine whether this mechanism of resistance applies to this case.

We also queried scRNA-seq data for mutations from the COSMIC (Catalogue of Somatic Mutations In Cancer) lung adenocarcinoma tier 1 mutations (Table S2), (Forbes et al., 2017; Shihab et al., 2015). Many of the mutations we identified had not been previously reported by the clinical-grade assay conducted on a patient’s tumor despite having been included in the clinical panel (Figure 2C; Figure S2I; Tables S2 and S3). Though this may reflect differences in biopsy technique or tumor clonality at the time of clinical testing, these results also demonstrate that clinical-grade bulk DNA-based testing may underestimate tumor heterogeneity.

To assess the clinical outcomes of patients harboring multiple oncogenic alterations and to determine the broader translational impact of our findings, we utilized the MSK-Impact NSCLC dataset (Zehir et al., 2017). Those patients whose tumor showed greater than or equal to 2 mutations from the tier 1 COSMIC mutation set detected in the scRNA-seq profiling (mutation high) had significantly lower overall survival (OS) compared to those patients whose tumor had less than 2 COSMIC tier 1 mutations (mutation low) (p < 0.01; Figure S2J). Thus, scRNA-seq analysis can provide increased granularity into cancer cell genomic heterogeneity and provides insight into the transcriptionally represented mutational landscape.

### Transcriptional Differences between TN and RD Cancer Cells Detected by scRNA-Seq Analysis Reveal Cell-State-Specific Biological Programs

**Figure S3:**
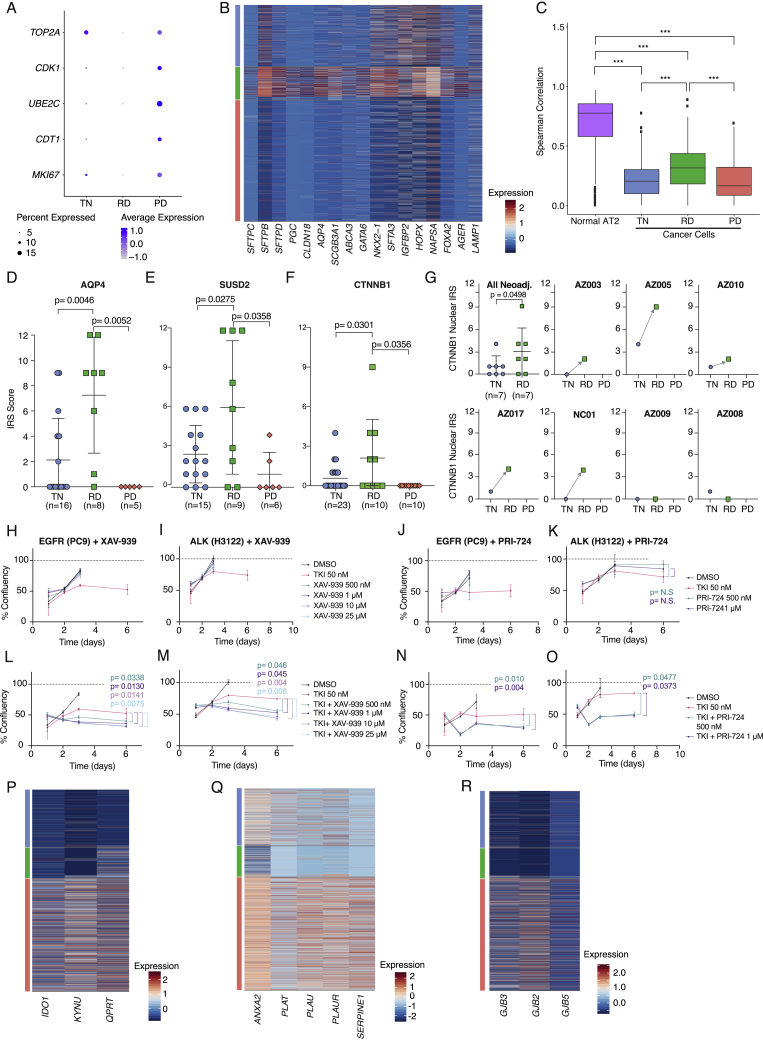
Related to Figure 3 (A) Dot plot of the relative expression of established cellular proliferation genes (x axis) across treatment time points (y axis). The color intensity scale reflects the average gene expression and the size scale indicates the number of cells expressing the gene within that treatment time point. Applying grouped, pairwise comparisons of treatment time points of the average scaled expression of all genes demonstrated significantly different expression (p < 0.0001) in all comparisons. (B) Heatmap showing the expression of genes in the alveolar signature. Cells are grouped by treatment time point. (C) Boxplot of Spearman correlations of cancer cells from all treatment time points and healthy AT2 cells to an external reference of healthy AT2 cells. Non-cancer AT2 cells from our dataset were more similar to the external, healthy AT2 cells than any of our cancer cells across all time points (mean ρ = 0.65, −0.10, 0.24, −0.19, for healthy AT2 cells, and TN, RD, PD cancer cells, respectively). ^∗∗∗^ indicates a p value < 0.001 (D-F) Immunoreactivity score (IRS) for membrane AQP4 (D), membrane SUSD2 (E), and nuclear CTNNB1 (F) across all time points. (G) Pairwise comparison of nuclear CTNNB1 IRS for a subset of patients receiving neoadjuvant TKI treatment prior to surgical removal of tumors, allowing for controlled, matched sample pairs at TN and RD treatment time points. Samples with AZ identifiers refer to patients with *EGFR* mutant NSCLC receiving neo-adjuvant osimertinib treatment. Sample with NC identifier refer to patient with *ROS1* fusion-positive NSCLC receiving neo-adjuvant crizotinib treatment. (H-O) High content microscopy screening of *EGFR* mutant PC9 cells and *ALK* fusion-positive H3122 cells showing treatment response to TKI in presence or absence of WNT/β-catenin inhibition. In comparison to DMSO control, upper panel (H-K) shows single agent treatment, lower panel (L-O) shows combinational treatment of TKI WNT/β-catenin inhibitors. Two WNT/β-catenin inhibitors have been tested, XAV-939 (H,I,L,M) and PRI-724 (J,K,N,O). Values are shown as percent confluency, with maximum cutoff for full well confluency (100%). *p* values are calculated for all end points (day 6) values compared to single agent TKI. Error bars represent mean ± standard error of the mean (SEM), n = 4 technical replicates. (P-R) Heatmaps showing the expression of genes within each signature (kynurenine, *SERPINE1*/plasminogen activation, and gap junction, respectively) grouped by treatment time point.

We hypothesized that defining the biological programs activated in cancer cells during therapy response may identify signaling pathways that promote adaptation and survival of cancer cells that comprise RD during initial treatment. We compared the transcriptional profiles of individual cancer cells obtained from tumor samples from TN to RD (Table S4) and focused on the 629 significantly (p < 0.05) upregulated genes in RD cancer cells as a proxy for evidence of pathway activation. We found numerous genes associated with cancer-associated pathways (Table S5). Importantly, we found that RD cancer cells expressed decreased proliferation marker genes compared to TN and PD, consistent with the expectation that during targeted treatment persisting cancer cells are generally less proliferative (Figure S3A) (Hsiao et al., 2019).

**Figure 3:**
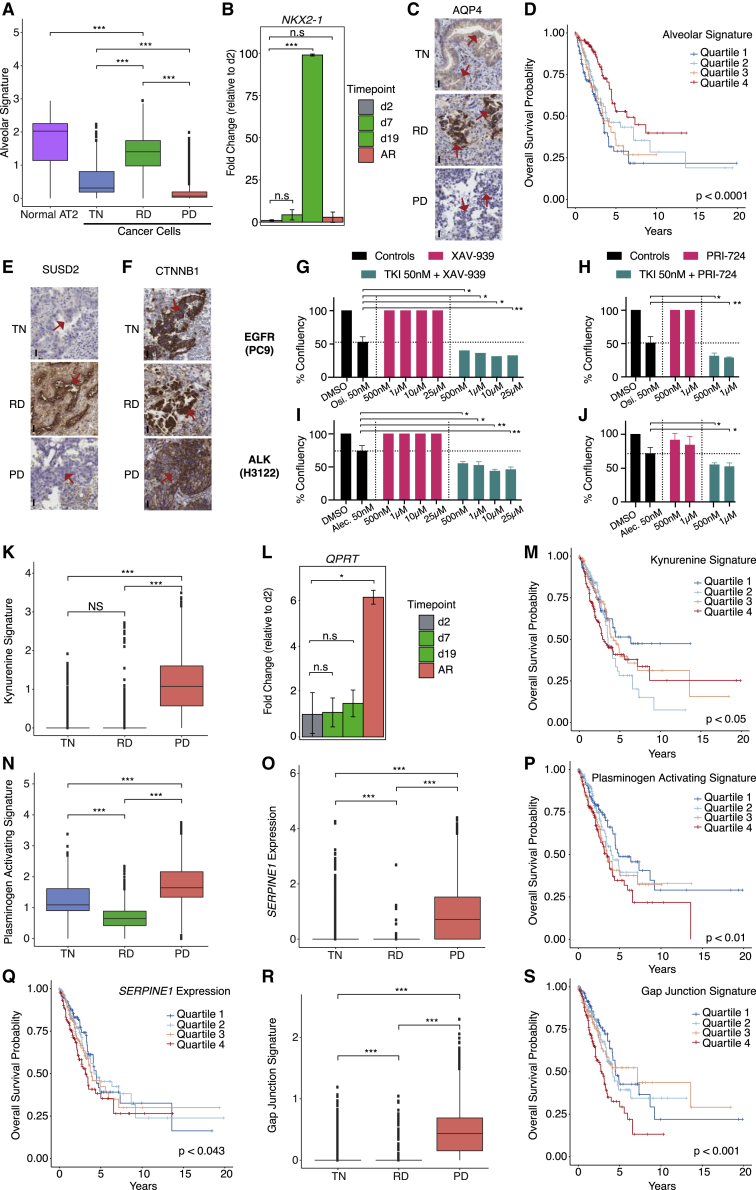
Differential Gene-Expression Analysis between Treatment Time Points Reveals Treatment Stage-Specific Transcriptional Signatures (A) Boxplots showing the expression level of the alveolar signature across treatment time points as well as non-cancerous AT2 cells from our cohort. ∗∗∗p < 0.001. (B) Fold-change expression of *NKX2-1* as quantified by RT-PCR in *EGFR* mutant PC9 cells after specified treatment duration (see STAR Methods), ^∗∗∗^p < 0.001. (C) Representative IHC images of TN, RD, and PD tumor tissue sections stained for AQP4 demonstrating increased expression at the RD time point. Red arrows indicate cancer cells of interest. Scale bars correspond to 50 μm. (D) Kaplan-Meier plot of the relationship between the alveolar signature and patient OS within the TGCA dataset. Patients were stratified by signature expression quartile (Q1 = 128, Q2 = 127, Q3 = 128, Q4 = 127), where Q1 is the lowest expression and Q4 is the highest expression. (E and F) Representative IHC images of TN, RD, and PD tumor tissue sections stained for SUSD2 (E) and CTNNB1 (F) demonstrating increased expression at the RD time point. Red arrows indicate example regions of interest. Scale bars correspond to 50 μm. (G–J) Treatment response upon inhibition of β-catenin activity in *EGFR* mutant PC9 cells and *ALK* fusion-positive H3122 cells. Relative viability is shown as percent confluency compared to DMSO control. PC9 cells were treated with XAV-939 (G) or PRI-724 (H) with or without the combination of 50nM osimertinib. H3122 were treated with XAV-939 (I) or PRI-724 (J) with or without the combination of 50 nM alectinib. p values were calculated for all end points (day 6) values compared to single agent TKI. Error bars represent mean ± standard error of the mean (SEM), n = 4 technical replicates. ^∗^p < 0.05, ^∗∗^p < 0.01. (K) Boxplots showing the expression levels of the kynurenine signature expression across different treatment time points. ∗∗∗p < 0.001. (L) Fold-change expression of *QPRT* as quantified by RT-PCR in PC9 cells after treatment with osimertinib as in (B) (see STAR Methods) (AR), ^∗^p < 0.05. (M) Kaplan-Meier plot of the relationship between the kynurenine signature and patient OS within the TGCA dataset. Patients were stratified by signature expression quartile (Q1 = 128, Q2 = 127, Q3 = 128, Q4 = 127), where Q1 is the lowest expression and Q4 is the highest expression. (N) Boxplot showing the expression levels of the plasminogen activation pathway signature across different treatment time points. (O) Boxplot showing the expression levels of the *SERPINE1* across different treatment time points. (P) Kaplan-Meier plot of the relationship between the plasminogen activating pathway signature and patient OS within the TGCA dataset, respectively. Patients were stratified by signature expression quartile (Q1 = 128, Q2 = 127, Q3 = 128, Q4 = 127), where Q1 is the lowest expression and Q4 is the highest expression. (Q) Kaplan-Meier plot of the relationship between *SERPINE1* expression and patient OS within the TGCA dataset, respectively. Patients were stratified by signature expression quartile (Q1 = 128, Q2 = 127, Q3 = 128, Q4 = 127), where Q1 is the lowest expression and Q4 is the highest expression. (R) Boxplot showing the expression levels of the gap-junction signature across treatment time points. (S) Kaplan-Meier plots of relationships between the gap-junction signature and patient OS within the TGCA dataset. Patients were stratified by signature expression quartile (Q1 = 128, Q2 = 127, Q3 = 128, Q4 = 127), where Q1 is the lowest expression and Q4 is the highest expression. See also Figures S3, S4, and S5 and Tables S2, S4, S5, and S6.

Interestingly, we identified an alveolar cell gene-expression signature composed of 17 established gene markers of alveolar cells (Vieira Braga et al., 2019; Wade et al., 2006) that showed significantly increased expression in RD versus TN time points **(**p < 0.0001; Figure 3A; Figure S3B; Table S2). Alveolar cells are comprised of alveolar type 1 (AT1) and type 2 (AT2) subtypes and form the lining of the lung alveoli. AT2 cells produce surfactants and can act as stem-like progenitor cells, which become active and proliferate in the setting of diverse types of lung injury and are suspected to be the cell of origin in oncogene-driven lung cancers (Desai et al., 2014; Hanna and Onaitis, 2013; Nabhan et al., 2018). AT1 cells are the dominant population in alveoli and mediate gas exchange and, when injured or dying, can release proliferation and regenerative signals (Desai et al., 2014). AT1 cells contain two population subtypes *HOPX*^+^/*IGFBP2*^+^ and *HOPX*^+^/*IGFBP2*^–^, the latter representing the cell population which maintains cellular plasticity and can proliferate as well as trans-differentiate into AT2 cells allowing for tissue regeneration after injury (Wang et al., 2018). The alveolar signature we detected in the cancer cells at RD includes both AT1- and AT2-associated genes (Table S2), including *AQP4*, *SFTPB/C/D*, *CLDN18*, *FOXA2*, *NKX2-1*, and *PGC* for AT2 cells (Desai et al., 2014; Liu et al., 2003; Nabhan et al., 2018; Wade et al., 2006; Xu et al., 2016; Zhou et al., 2018) and *AGER*, *HOPX*, and *IGFBP2* for AT1 cells (Nabhan et al., 2018; Serveaux-Dancer et al., 2019; Figure S3B). Additionally, the alveolar cell state we identified in cancer cells was not derived from mis-annotated non-cancer alveolar cells within our cancer cell populations (Figure S3C).

We validated the activation of the alveolar cell signature at RD using orthogonal approaches. First, we used an established preclinical model consisting of patient-derived *EGFR* mutant NSCLCs (PC9) (Lee et al., 1985) to develop analogs of the TN, RD, and PD clinical states. Using RT-PCR, we measured the expression of *NKX2-1*, a hallmark alveolar cell signature gene upregulated in RD clinical samples and found a significantly higher (p < 0.001) expression in the persister state cells compared to control and acquired resistance state cells (Figure 3B**)**. This suggests that the alveolar signature identified from the clinical scRNA-seq analysis can be reproduced under controlled conditions *in vitro*. Furthermore, immunohistochemical (IHC) analysis showed induction of AQP4 protein expression, another marker of the alveolar cell signature, at the plasma membrane of RD clinical samples compared to both TN and PD clinical samples (Figure 3C; Figure S3D).

We tested whether the alveolar signature is a clinically relevant biomarker of patient survival in the TCGA lung adenocarcinoma bulk RNA-seq dataset (https://www.cancer.gov/about-nci/organization/ccg/research/structural-genomics/tcga, Cancer Genome Atlas Research et al., 2013). We found a significant (p < 0.0001) association between high expression of our alveolar signature in these tumors and improved patient OS when compared to patients whose tumors showed a lower alveolar expression signature (Figure 3D; Table S6).

These findings support the assertion that there is a distinct alveolar gene-expression signature characterizing RD cancer cells, associated with improved patient survival. A plausible model is that the identified alveolar signature that is activated in RD cancer cells reflects a cell injury and repair signature, reminiscent of non-cancerous AT1 and AT2 cells (Nabhan et al., 2018; Wang et al., 2018). Increased expression of this signature could lead to repair and escape of cell death during treatment to support cancer cell persistence, while at the same time constituting a less aggressive malignant state. This is consistent with the notion that RD represents a persister cell state observed in preclinical models of slow-cycling cancer cells that survive without rapid proliferation (as in Figure S3A), as a prelude to the onset of aggressive tumor progression upon absolute drug resistance (Hata et al., 2016).

The molecular details of the alveolar and cell injury repair signature are notable. In our RD cohort, the WNT/β-catenin-associated pathway genes *SUSD2* and *CAV1* exhibited increased expression (Table S5). We used IHC analysis of both SUSD2 and CTNNB1 (β-catenin) protein expression (Figures S3E and S3F) to validate the observed transcriptional changes. In agreement with our scRNA-seq findings, we found significantly increased membrane SUSD2 and significantly increased nuclear CTNNB1 (β-catenin) in the RD state compared with both TN and PD. Additionally, the comparison of nuclear localization of CTNNB1 in a unique series of paired TN and RD samples obtained from *EGFR*(AZ) or *ROS1*(NC) patients treated with neoadjuvant TKI on one of two clinical trials (osimertinib: NCT03433469 or crizotinib: NCT03088930) is shown in Figure S3G. SUSD2 is an activated downstream target of the WNT pathway (Umeda et al., 2018; Xu et al., 2018b), while *CAV1* can promote nuclear localization of β-catenin (*CTNNB1*) and transcriptional activation of the WNT/β-catenin pathway (Yu et al., 2014). In NSCLCs, the WNT/β-catenin signaling pathway contributes to tumorigenesis (Juan et al., 2014; Nakayama et al., 2014; Pacheco-Pinedo et al., 2011), repair, and regeneration after cell injury (Huch et al., 2013; Tammela et al., 2017). The self-renewal and injury response in AT2 cells specifically can utilize the WNT/β-catenin signaling pathway (Nabhan et al., 2018; Stewart, 2014). Additionally, in *EGFR* mutant NSCLC activation of the WNT/β-catenin pathway may limit EGFR inhibitor response and may promote survival of a persister cell population during EGFR inhibitor therapy *in vitro* (Arasada et al., 2018; Blakely et al., 2017; Casás-Selves et al., 2012; Nakayama et al., 2014). Overall, the RD state is characterized by signals of cellular injury and survival, which act, in part, through the WNT/β-catenin pathway, which may be therapeutically targetable (Krishnamurthy and Kurzrock, 2018).

The clinical data suggest that WNT/β-catenin activation is engaged early during treatment to facilitate the development of RD and drug tolerant persister cells during primary EGFR or ALK targeted therapy. To further explore the therapeutic potential of the WNT pathway findings, we utilized patient-derived PC9 cells as an *EGFR* mutant NSCLC model and H3122 cells as a model for *ALK* fusion-driven NSCLCs. We tested the hypothesis that upfront blockade of WNT/β-catenin signaling together with oncogenic *EGFR* or *ALK* would decrease the number of cells surviving initial treatment and increase the depth of response from the outset of therapy. Parental cells were treated with an IC_50_ (inhibitor concentration yielding a 50% decrease in cell number) dose of the appropriate EGFR or ALK TKI (osimertinib or alectinib, respectively). Two different WNT/β-catenin pathway inhibitors XAV939 and PRI-724 in four previously reported concentrations or combination therapy thereof were tested. Our *in vitro* results support our hypothesis by demonstrating that the upfront inhibition of the WNT/β-catenin pathway in combination with the cognate TKI led to a significant and dose dependent decrease in cell confluency and increased depth of response (Figures 3G–3J; Figures S3H–S3O).

### Transcriptional Differences between TN and PD Cancer Cells Reveal Immune Modulation and Cellular Invasion as Key Features of Cancer Progression

When comparing cancer cells from TN and PD samples, we found 901 differentially upregulated genes in PD cancer cells (Table S4). Within those genes, we identified genes involved in the kynurenine pathway and multiple genes and pathways associated with oncogenesis and inflammation (Table S5).

We observed a significant (p < 0.0001) increase in the expression of *IDO1*, *KYNU*, and *QPRT* genes involved in the kynurenine pathway, in PD and TN cancer cells (Figure 3K; Figure S3P; Table S2) Expression of these genes can result in immunosuppressive behavior (Triplett et al., 2018) indicating that cancer cells within PD tumors may directly inhibit the activity of the immune system. The identification of this pathway as a mediator of immune suppression within PD tumors has important potential therapeutic implications, as *IDO1* is upregulated in many cancers (Cheong and Sun, 2018; Hornyák et al., 2018; Liu et al., 2018). Multiple clinical trials have attempted to block this pathway using IDO1 inhibitors as a monotherapy as well as in combination with immune checkpoint inhibitors or hormone therapy (Ricciuti et al., 2019), albeit with limited success. *QPRT* also exhibited increased expression (p < 0.05) specifically at acquired resistance of EGFR inhibitor treatment (i.e., the analog of clinical PD) in our *in vitro* model using PC9 cells (Figure 3L), reinforcing the assertion that this pathway is indicative of cancer progression under the selective pressure of treatment.

To further demonstrate the clinical relevance of the kynurenine pathway, we again used the TCGA lung adenocarcinoma RNA-seq dataset. Higher tumor expression of this signature was a biomarker of worse OS in patients (p < 0.05; Figure 3M; Table S6). This is consistent with the notion that activation of this pathway leads to immunosuppression and an inability of the immune system to effectively surveil and eradicate cancer cells.

### scRNA-seq Profiles of Cancer Cells Change from RD to PD

We compared cancer cells from RD and PD patient samples to elucidate the differences that occur during the outgrowth of PD from RD and found a total of 2,182 genes which had significantly (p < 0.001) increased expression in either RD or PD (N_RD_ = 1,121, N_PD_ = 1,061) (Table S4). Among the differentially overexpressed genes at RD were genes associated with the alveolar cell signature, cell growth, differentiation, cell motility, and tumor suppression (Table S5). RD cancer cells overexpress surfactant genes (*SFTPB/C/D* and *SFTA3*), which are part of the alveolar cell signature (Figure 3A; Figure S3B) (Desai et al., 2014; Treutlein et al., 2014; Wang et al., 2018). Furthermore, *NKX2-1* and *NFIX* were overexpressed in RD cancer cells and are associated with decreased cell motility (Ge et al., 2018; Rahman et al., 2017; Winslow et al., 2011). Low expression of *NKX2-1* leads to loss of differentiation and enhanced tumor seeding ability (Winslow et al., 2011). The collective findings arising from this and the previous RD cancer cell analyses suggest that an injury-repair and regenerative cell state may promote cancer cell indolence, increased tumor control, and improved clinical outcomes.

By contrast, PD cancer cells differentially overexpressed genes associated with invasion, cell-to-cell communication, differentiation, and immune modulation (Table S5). Several genes in the plasminogen activation pathway were significantly overexpressed (*ANXA2*, *PLAT*, *PLAUR*, *PLAU)* (Figure 3N) along with the plasminogen inhibitor *SERPINE1* (PAI1*)* (p < 0.0001, Figure 3O; Figure S3Q*)*. *ANXA2* and *PLAUR* are the receptor proteins in the plasminogen activation cascade and involved in inflammation, angiogenesis, invasion, and metastasis, via degradation of the extracellular matrix (Kubala et al., 2018; Zhu et al., 2017). Signaling is initiated when *ANXA2* or *PLAU* binds to *PLAT* (uTa) or *PLAU* (uPa), respectively. Plasminogen is then degraded to plasmin through the activity of *PLAT* and/or *PLAU* leading to activation of metalloproteinases and degradation of fibrin. *SERPINE1* shows increased expression in a number of cancer subtypes and plays important roles in cell adhesion, invasion, tumor vascularization, radio-resistance, and immunosuppression (Kubala et al., 2018; Zhu et al., 2017). High expression of the plasminogen activation signature correlated with worse patient OS (p < 0.01) within the TCGA lung adenocarcinoma RNA-seq dataset and cohort (Figure 3P; Table S6). Similarly, in this independent dataset high expression of *SERPINE1* was associated with worse OS (p < 0.05) (Figure 3Q; Table S6). EGFR inhibitor therapy can induce expression of *SERPINE1* and *EGFR* mutant patients with greater than 2-fold induction of SERPINE1 (PAI1) plasma levels during EGFR inhibitor treatment demonstrated shorter progression-free survival (Arasada et al., 2018). Collectively, our scRNA-seq findings shed light on the clinical relevance and potential role of the plasminogen activation cascade in inferior clinical outcomes and targeted therapy resistance.

Additionally, we found several gap-junction proteins differentially overexpressed in PD cancer cells compared to RD cancer cells (p < 0.0001, Figure 3R; Table S2). Gap-junction proteins (e.g., connexins) are integral membrane proteins that allow for cytosolic exchange of ions, metabolites and secondary messengers between cells (Aasen et al., 2016; Sinyuk et al., 2018). While some have been identified as tumor suppressors, we found that high expression of *GJB2/3/5* (Figure S3R; Table S6) was linked to worse survival in the TCGA lung adenocarcinoma RNA-seq dataset (p < 0.001) (Figure 3S). These collective findings suggest a pro-tumor effect not only in our cohort but also in NSCLCs more generally.

**Figure S4:**
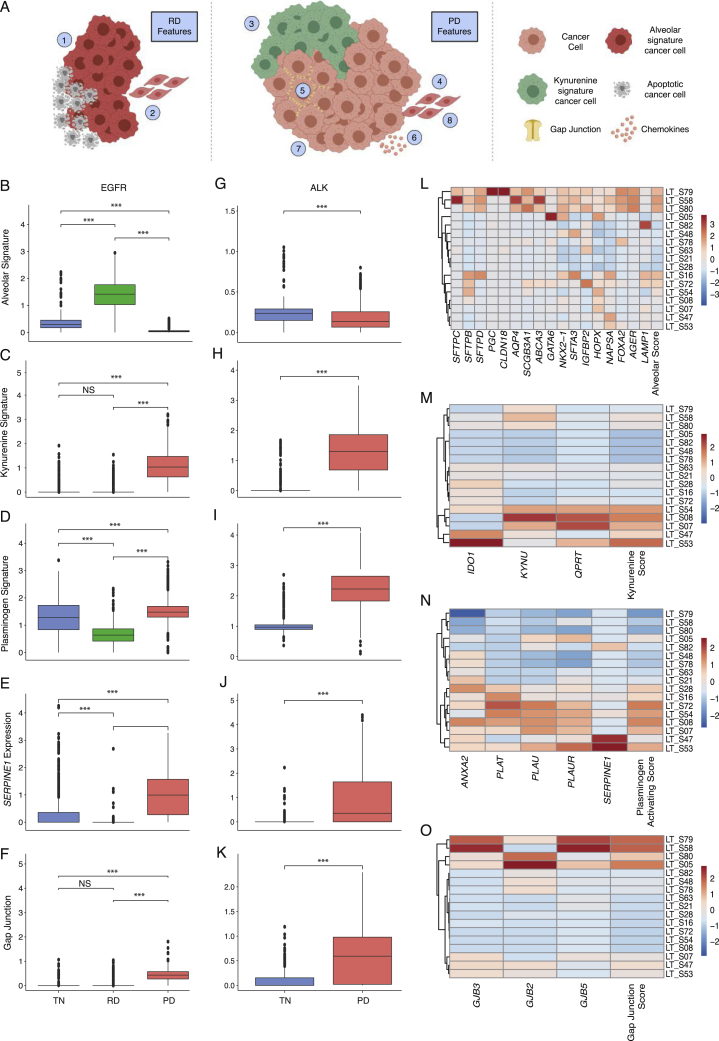
Related to Figure 3 (A) Graphical summary of cancer cell expression changes across treatment time points. RD features include (1) Alveolar signature, and (2) various RD specific invasive signaling pathways. PD features include: (3) kynurenine signature, (4) plasminogen activation and *SERPINE1* signatures, (5) gap junction proteins, (6) expression of pro-inflammatory chemokines, (7) loss of tumor suppressor genes, and (8) various PD specific invasive signaling pathways. (B-F) Boxplots of pathway signature changes (alveolar, kynurenine, plasminogen activating, *SERPINE1*, and gap junction, respectively) across treatment time points within only *EGFR* mutant cancer cells (^∗∗∗^ indicates a p value < 0.0001). (G-K) Boxplots of pathway signature changes (alveolar, kynurenine, plasminogen activating, *SERPINE1*, and gap junction, respectively) across treatment time points within only *ALK* cancer cells (^∗∗∗^ indicates a p value < 0.0001). (L-O) Heatmap of sample average expression with PD only cancer cells for each cancer derived signature gene (alveolar, kynurenine, plasminogen activating/*SERPINE1*, and gap junction, respectively).

Within cancer cells, we identified a rich complexity of clinically relevant, expressed mutations that may impact therapy response. Furthermore, evaluation of transcriptional profiles of individual cancer cells across different treatment time points identified several clinically relevant cell-state changes (Figure S4A). We found that the identified treatment time point signatures largely persist irrespective of the type of oncogenic driver mutation (*EGFR* or *ALK*) (Figures S4B–S4K) and of biopsy site (primary or metastatic) (data not shown). While we found these cancer cell signatures are robust, it is important to acknowledge that there is patient heterogeneity among samples (Figures S4L–S4O).

### Longitudinal scRNA-seq Analysis of an Individual Patient’s Tumor during Treatment

**Figure S5:**
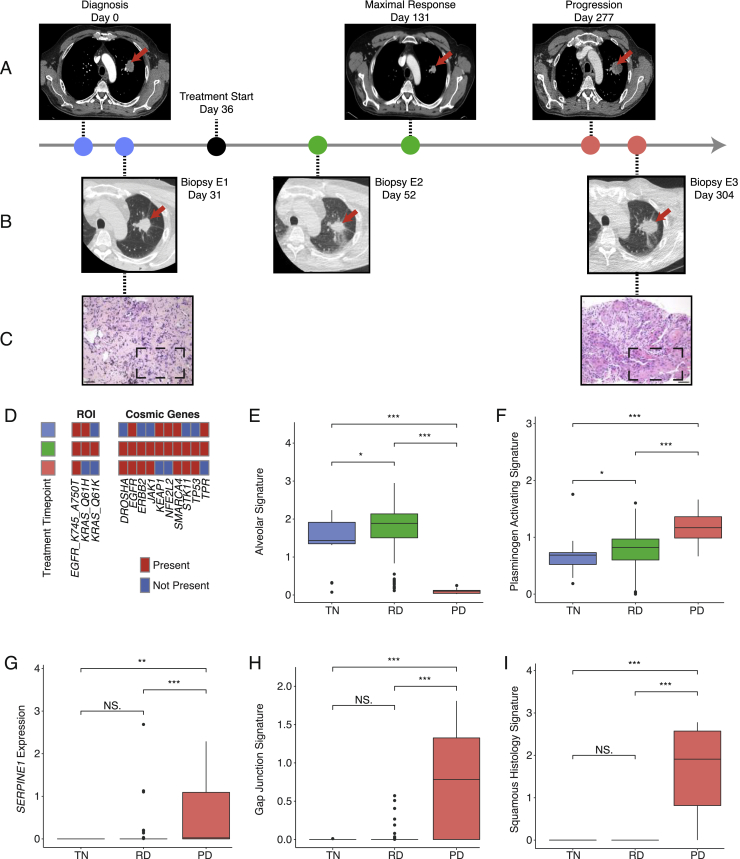
Related to Figure 3 (A, B, C) Longitudinal timeline of patient treatment, (A) Chest CT scan at each clinical evaluation time point, (B) Biopsy time point with procedural CT scan, (C) Hematoxylin and eosin (H&E) staining from treatment naive and progression time points demonstrating adenocarcinoma and squamous cell carcinoma, respectively, scale bar indicates 50 μm. (D) Heatmap of mutation state in clinical driver and a subset of COSMIC tier 1 mutated genes (displayed COSMIC tier 1 mutations occur in at least two out of three samples). Color red indicates the presence of mutation whereas color blue indicates no presence of mutation. (E-I) Boxplots of pathway signature changes (alveolar, plasminogen activating, *SERPINE1*, gap junction and squamous histology, respectively) across treatment time points.

Obtaining consecutive clinical tumor biopsies from individual advanced-stage lung cancer patients before and during treatment is challenging given that most tumors regress by 50% or greater, albeit incompletely, during TKI treatment (Camidge et al., 2019; Soria et al., 2018). Nevertheless, we obtained samples from the same primary tumor site from 3 treatment time points from a patient (TH226) whose tumor contained a standard *EGFR* exon 19 deletion oncogenic mutation and was treated with the EGFR inhibitor osimertinib (Figures S5A–S5C). In all 3 biopsies, we identified by scRNA-seq RNA expression of the *EGFR* exon 19 driver mutation in the cancer cells and several other mutations of interest (Figure S5D).

When comparing TH226 to the rest of the scRNA-seq dataset, we found overlapping differentially expressed genes and signatures (Table S4; Figures S5E–S5H). Intriguingly, we also found numerous genes associated with squamous cell differentiation (*KRT16*, *KRT14*, *KRT6A*, *KRT5*, *CLCA2*, *PKP1*, *ANXA8*, *DSG3)* overexpressed at PD compared to TN and RD time points (p < 0.0001, Figure S5I, Tables S4 and S5) (Ben-Hamo et al., 2013; Chao et al., 2006; Goodwin et al., 2017). This is particularly interesting given that the patient’s lung tumor biopsy at PD demonstrated a histologic shift to squamous cell carcinoma from that of prior biopsies that showed pure adenocarcinoma histology (Figure S5C). Histologic transformation to squamous cell carcinoma is a mechanism of EGFR inhibitor resistance in *EGFR* mutant NSCLCs (Izumi et al., 2018; Jukna et al., 2016). Thus, scRNA-seq has the power to provide a high resolution, gene and pathway level view of biological and histological plasticity that arises during cancer drug treatment.

### Inversion of Myeloid and Lymphoid Infiltration within the TME at Progressive Disease Compared to RD

**Figure 4:**
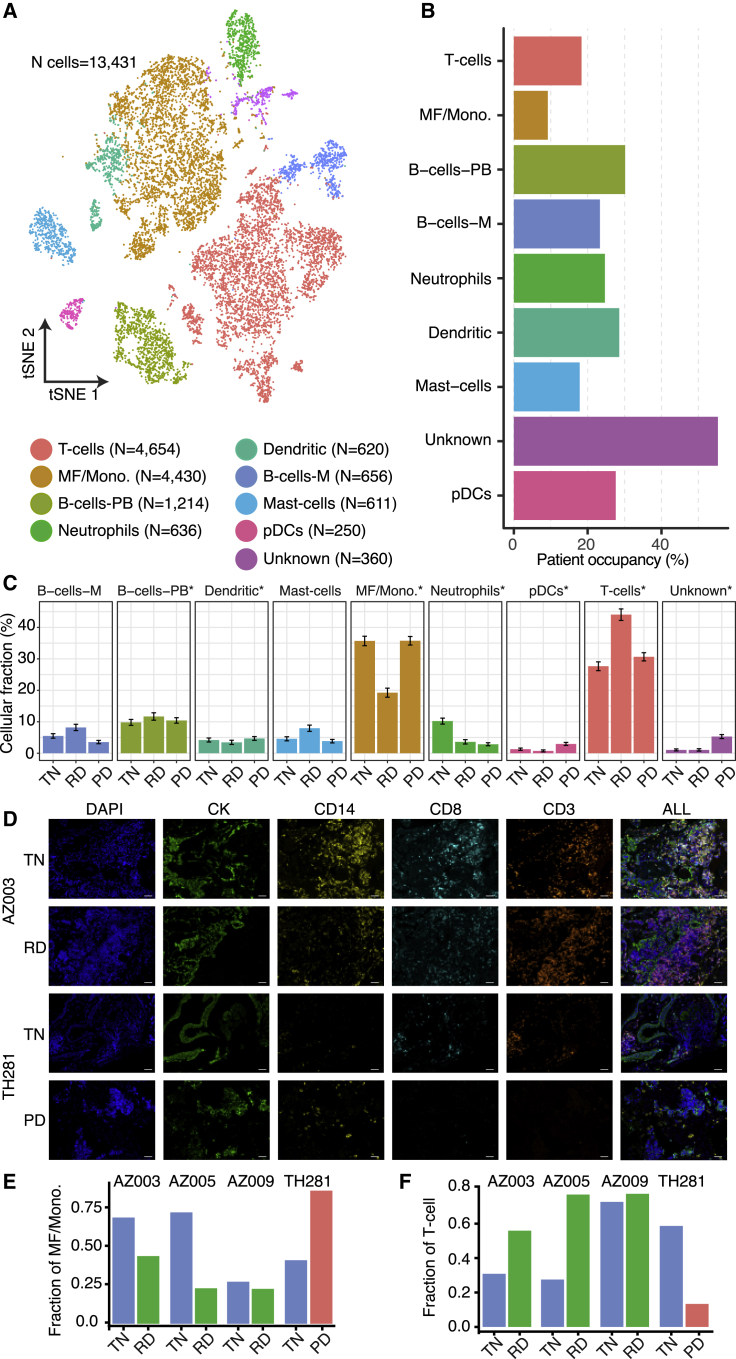
Changes in the Composition of the Tumor Microenvironment within Each Tumor (A) t-SNE plot of all immune cells colored by immune cell type. (B) Patient occupancy for each immune cell type. (C) Fractional changes for each immune cell type across the three treatment states. Error bars indicate the 95% confidence interval for the calculated relative frequencies. ^∗^p < 0.01 using a chi-square test of independence. (D) Representative *in situ* immunofluorescence images of changes from TN to RD and TN to PD in tumor tissue sections from two separate samples at two separate time points; AZ003 (TN and RD), TH281 (TN and PD). Scale bars correspond to 50 μm. (E) Quantification of fractional changes of macrophages across treatment time points from the images in (D) and Figure S5F. (F) Quantification of fractional changes of T-cells across treatment time points from the images in (D) and Figure S5F. See also Figure S6.

We next addressed the evolution of the TME during targeted treatment. Immune cells (n = 13,431) were clustered and annotated (Figure 4A; Tables S1 and S4). In contrast to clusters of cancer cells, which were predominantly patient specific (Figures S2C and S2D), immune cell-type clusters showed low patient occupancy (Figure 4B). This is consistent with the expectation of finding common immune cell phenotypes across patients and samples.

**Figure S6:**
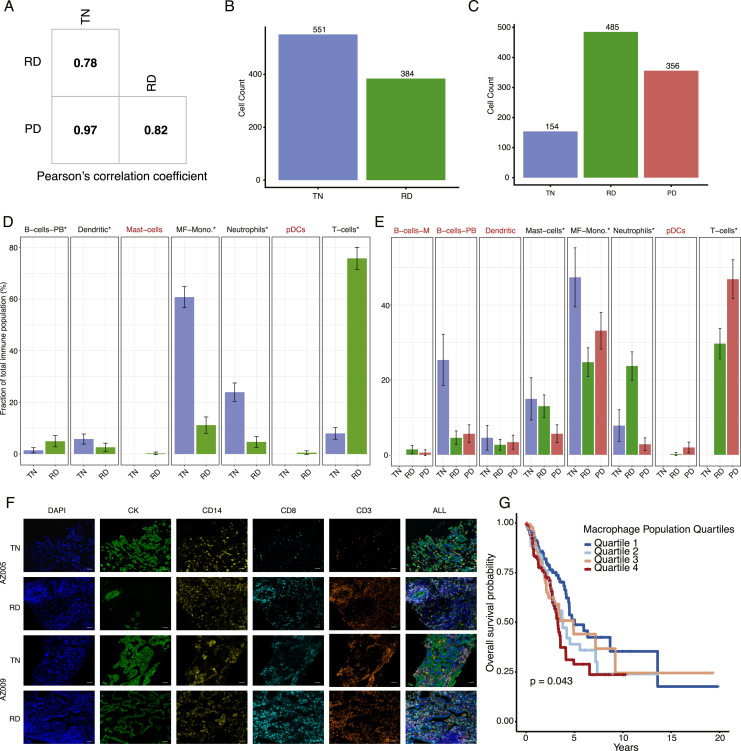
Related to Figure 4 (Α) Pairwise Pearson correlations between each treatment group’s immune cell compositions which corresponds to the fraction of each immune cell type’s abundance in the total immune cell population. (B) Total immune cells for each biopsy of patient TH266. (C) Total immune cells for each biopsy of patient TH226. (D) Fraction of each immune cell subtype for the two biopsies of patient TH266. Error bars indicate the 95% confidence interval for the calculated relative frequencies. Asterisks next to the title of each cell type indicate significance (p < 0.01) when using a chi-square test of independence. Titles of non-significant cell types are colored red and lack an asterisk. (E) Fraction of each immune cell sub-type for the three biopsies of patient TH226. Error bars indicate the 95% confidence interval for the calculated relative frequencies. Asterisks next to the title of each cell type indicate significance (p < 0.01) when using a chi-square test of independence. Titles of non-significant cell types are colored red and lack an asterisk. (F) Representative *in situ* immunofluorescence images from two patients with matched samples at different treatment time points, demonstrating fractional changes in the immune populations of macrophages and T cells. Scale bars correspond to 50 microns. (G) Kaplan-Meier plot of deconvoluted TCGA lung adenocarcinoma data showing the relation between OS and the fraction of macrophages for each patient. Patients were stratified by high and low macrophage fraction.

We compared the immune cell composition across all 3 time points, expressed as the correlation between fractional immune cell abundance vectors. The immune composition within RD was the most dissimilar from the other two treatment states (r = 0.78 versus TN samples, r = 0.82 versus PD samples, Pearson’s correlation coefficient) (Figure S6A). Across all treatment time points, T cells and macrophages were the dominant cell populations and demonstrated an inversion in relative abundance during tumor response and resistance to treatment, a finding we examined further as described below (Figure 4C). T cells comprised a larger fraction of all immune cells within the TME at RD compared to TN or PD samples (27% T cells TN, 46% RD, 31% PD). Macrophage infiltration followed the inverse pattern, with a decrease in macrophages at RD compared to TN and PD (37% macrophages TN, 21% RD, 37% PD).

In 2 patients, we examined immune cells from available matched tumor biopsies obtained at different treatment time points (TH226 and TH266, Figures S6B and S6C, respectively). In 2 tumor biopsies available for patient TH266, both macrophages and T cells showed reduction in the fraction of macrophages and an increase in the fraction of T cells from TN to RD, findings which match the entire cohort (Figure S6D). TH226 exhibited a similar pattern with the fraction of macrophages decreasing at RD after initiation of treatment and increasing again at PD (Figure S6E). We validated our findings on tissue samples using immunofluorescence staining (Figures 4D–4F; Figure S6F). Additionally, we deconvoluted TCGA bulk transcriptome data for NSCLCs into fractions of immune cells types (see STAR Methods) and found that TCGA samples with high fractions of macrophages had significantly worse OS (p < 0.01) (Figure S6G). This supports the clinical relevance of our observations and is consistent with prior reports associating macrophage infiltration with poor prognosis in patients who undergo surgical resection of early-stage NSCLCs (Chen et al., 2005; Zhang et al., 2011), as well as with worse progression-free survival during EGFR TKI therapy (Chung et al., 2012).

These findings are particularly intriguing given their similarity to melanoma tumors treated with PD-1 inhibitor (Riaz et al., 2017), albeit here in the distinct context of oncoprotein-targeted therapy in lung cancer. Specifically, an increase in the number of CD8^+^ T cells and natural killer (NK) cells and a decrease in M1 macrophages were observed in melanoma during PD-1 inhibition. There may be common responses in NK/T cells and macrophages during treatment across different tumor histologies and treatments. Hence, conserved approaches to targeting RD across different cancer subtypes and therapeutic modalities may exist, an area for future investigation.

### An IDO1-Expressing Macrophage Population Is Enriched at PD

**Figure S7:**
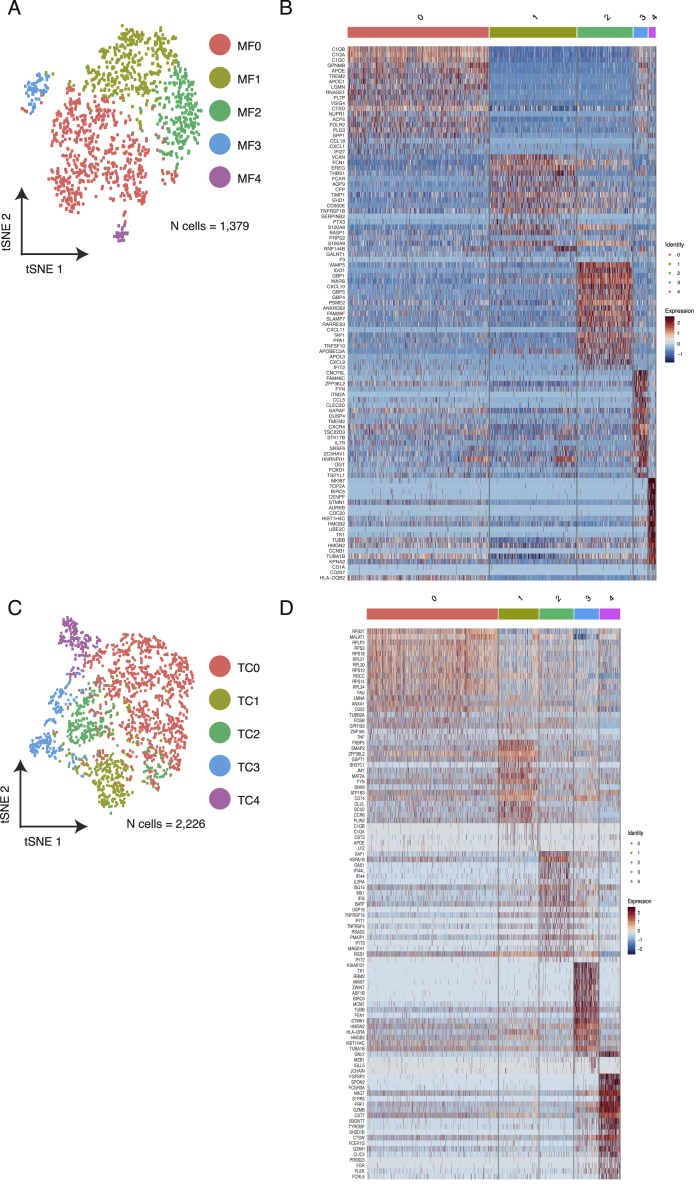
Related to Figure 5 (Α) t-SNE plot of all lung-derived macrophage cells. (B) Heatmap showing the expression level of the top 10 differentially expressed genes for each macrophage cluster. (C) t-SNE plot of all lung-derived T cells. (D) Heatmap showing the expression level of the top 10 differentially expressed genes for each T cell cluster.

**Figure 5:**
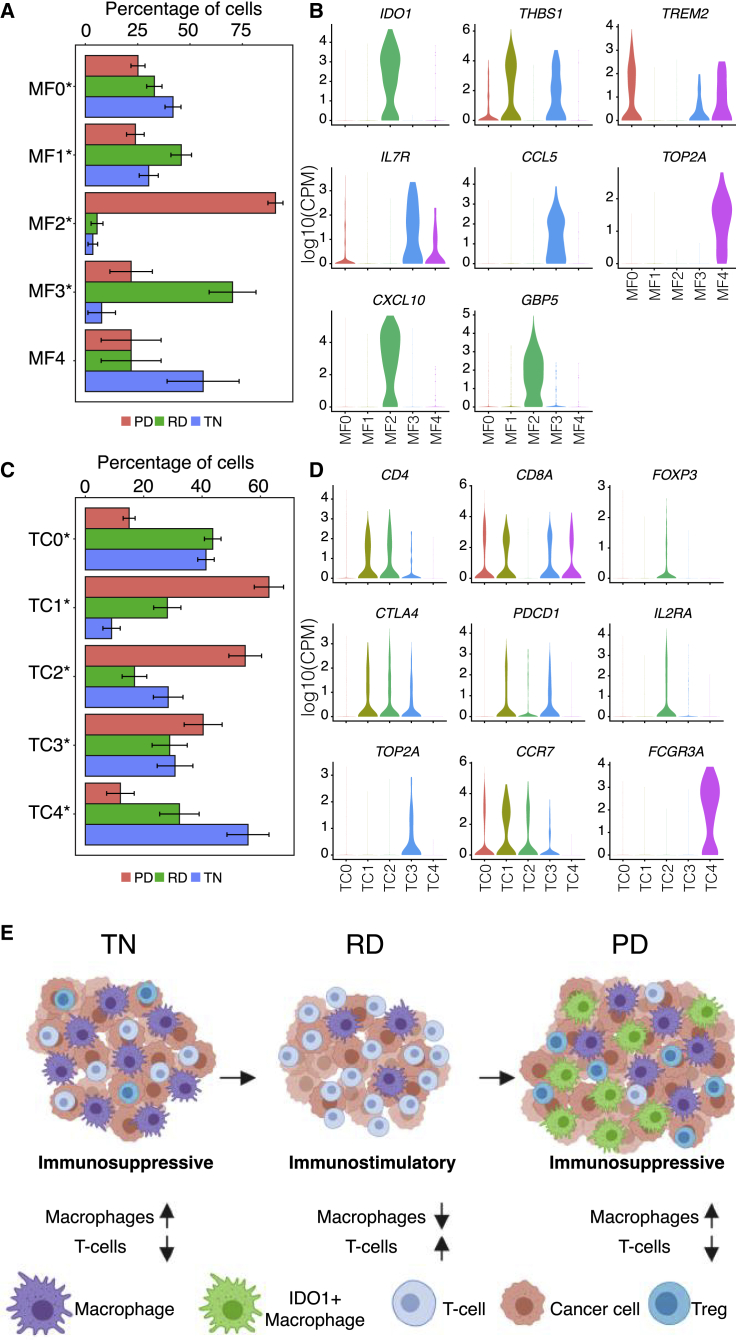
Immune Cell Subpopulations Demonstrate Unique Transcriptional Profiles within Each Treatment Time Point (A) Fraction of cells belonging to each treatment stage for each macrophage cluster in Figure S6. Error bars indicate the 95% confidence interval for the calculated relative frequencies. ^∗^p < 0.01 using chi-square test of independence. (B) Violin plots showing the expression level distribution of notable individual genes. (C) Fraction of cells belonging to each treatment stage for each T cell cluster in Figure S6. Error bars indicate the 95% confidence interval for the calculated relative frequencies. ^∗^p < 0.01 using chi-square test of independence. (D) Violin plots showing the expression level distribution of notable individual genes. (E) Graphical summary of immune microenvironment changes across treatment time points. See also Figure S7 and Tables S4 and S5.

Macrophages from lung tumor biopsies (n = 1,379) were clustered into 5 distinct groups (Figure S7A) followed by differential gene expression in each resulting cluster (Figure S7B; Table S4). In addition, we calculated the fraction of cells originating from each of the three treatment groups in each of the 5 macrophage clusters (Figure 5A).

Cluster MF0, which was slightly enriched in TN cells, was characterized by expression of genes associated with an immunosuppressive phenotype (*C1QC*, *GPNMB*, *APOE*, *TREM2*, *FOLR2*) (Cochain et al., 2018; Zhang et al., 2019; Zhou et al., 2017) (Figure 5B; Figure S7B). Clusters MF1 and MF3 were enriched at RD. Macrophage cluster MF1 expressed features associated previously with tumor-infiltrating myeloid derived suppressor cells (*FCN1*, *VCAN*, *S100A8*, *S100A9*) (Zhang et al., 2019) and with *THBS1* and *PTX3*, which are associated with resolution of inflammation, wound healing, and with inhibition of IL-1β (Bouhlel et al., 2007; Faz-López et al., 2016; Martinez and Gordon, 2014; Puig-Kröger et al., 2009; Shiraki et al., 2016; Stein et al., 2016; Zhang et al., 2019) (Figure 5B; Figure S7B). Cluster MF3 expressed genes associated with pro-inflammatory response to tissue damage (*CLEC2D*, *IL7R*, *OGT)* and with promoting inflammatory signaling (*FYN*, *DUSP4*, *FOXO1*) (Bao et al., 2018; Fan et al., 2010; Lai et al., 2020; Mkaddem et al., 2017; Moriwaki and Asahi, 2017). Macrophages in this cluster express *CCL5*, a cytokine that has previously been associated with promotion of residual HER2^+^ breast cancer survival following HER2-targeted therapy (Walens et al., 2019). Cluster MF4 consisted of proliferating myeloid cell populations (*TOP2A*, *MKI67*) and did not significantly differ between groups.

Macrophages at PD were overrepresented in group MF2 (Figure 5A) and expressed pro-inflammatory cytokines *CXCL9*, *CXCL10*, and *CXCL11* (Figure 5B; Figure S7B), which favor lymphocyte recruitment into the TME (Nagarsheth et al., 2017). Top differentially expressed genes in this population also included the guanylate-binding family proteins *GBP1* and *GBP5*, which are induced in IFN-γ-activated macrophages and promote inflammatory signaling within the innate immune system via inflammasome assembly (Shenoy et al., 2012) (Figure 5B; Figure S7B). Despite the expression of pro-inflammatory genes within the MF2 macrophages, the top differentially expressed gene within this group of PD-specific macrophages was *IDO1* (Figure 5B). *IDO1* is induced by inflammation within the TME and promotes a tolerogenic environment through immunosuppressive myeloid cell populations, regulatory T cell (Treg) differentiation, and an immunosuppressive cytokine milieu (Munn and Mellor, 2016).

### An Immunosuppressive T Cell Phenotype Is Predominant within the TME at PD

T cells and NK cells (n = 2,226) were analyzed in the same manner as macrophages and resulted in 5 distinct T/NK cell populations (Figure 5C; Figure S7C). These included two populations (TC0, TC4) enriched in TN samples and 2 populations (TC1, TC2) enriched at PD (Figures 5C and 5D; Figure S7D). There was a high overall fraction of T cells in RD tumors (Figure 4C), and there was no single T cell cluster that demonstrated an excess of T cells in RD (Figure 5C).

Both TN and PD T cells demonstrated a relative decrease in T cell infiltration (Figure 4C). The T cells which were present in the TN state were enriched for T cell populations TC4 and TC0. TC4 expressed markers consistent with a natural killer (NK) or natural killer T cell (NKT) phenotype, including NK cell markers (*KIR2DL3*, *FCGR3A*) as well as moderate expression of T cell markers (*CD3*, *CD8)*. TC0 reflected a naive-like CD8^+^ phenotype with expression of *CCR7*, *IL7R*, and *SELL* (Figure 5D; Figure S7D) (van der Leun et al., 2020). While overall T cell infiltrate remained limited at PD (Figure 4C), there was relative enrichment for T cell phenotypes with immunosuppressive features, including T cell clusters TC1 and TC2 (Figure 5C). TC1 was identified as a T cell cluster with a dysfunctional or exhausted phenotype, characterized by expression of the inhibitory receptors *PDCD1* (which encodes for the PD-1 protein) and *CTLA4 (**Wherry and Kurachi*, *2015**)* (Figure 5D). TC2 was composed of Treg cells (expressing *FOXP3*, *IL2RA*). Consistent with a relatively immunosuppressive environment, there was additionally reduction at PD in infiltration by the NK/NKT cell cluster TC4.

Tumor biopsies obtained at RD revealed the presence of a more pro-inflammatory, “hot,” TME, which was absent in TN or PD biopsy samples as manifested by increased overall proportion of T cells and reduced infiltration by regulatory T cells (TC2) (Figure 5E). Compared to the PD state, at RD there were fewer dysfunctional T cells (TC1) and greater NK/NKT cell (TC4) infiltration (Figure 5D; Figure S7D). A population of proliferating tumor infiltrating T cells was shared across all treatment states (TC3) and was slightly enriched within PD samples. This T cell population was characterized by both cytotoxic phenotypes (*CD8*, *GZYMB*) and *PDL1*/*CTLA4* expression and may reflect a pre-dysfunctional cytotoxic T cell population (van der Leun et al., 2020).

In summary, both the TN and PD TME were characterized by the relative predominance of macrophage over T cell infiltration; however, the phenotypic characteristics of these infiltrating immune cells differ between the two groups. At PD, there was infiltration by an *IDO1*^+^ macrophage population, of proliferating regulatory T cells, and of dysfunctional T cells which were minimally present at TN and RD. In contrast, the TN state was characterized by a predominance of more classically immunosuppressive M2-like macrophages (Sica et al., 2008). By distinction, in RD there was increased infiltration of T cell populations without dysfunctional or immunosuppressive gene-expression patterns and decreased immunosuppressive macrophage infiltration (Figure 5E).

## Discussion

There remains an incomplete catalog of single-cell transcriptional data that can be used to understand cell states and the therapy-induced evolution of biological heterogeneity of diseases such as cancer, particularly for advanced-stage solid malignancies. Patients with metastatic disease do not routinely receive surgical resection as part of their treatment. Thus, techniques for single-cell profiling that require larger amounts of tissue are not suitable for the interrogation of tissue samples from metastatic disease (Lambrechts et al., 2018; Schelker et al., 2017). Our scRNA-seq analyses of advanced-stage NSCLC biopsies obtained at different treatment time points from patients elucidate the rich mutational and transcriptional diversity within individual tumor samples and the dynamic changes in the transcriptional profiles of cancer cells and the TME composition during treatment. Our findings provide a roadmap that highlights the underlying cellular ecosystem and mechanisms that can inform efforts to better treat oncogene-driven cancers. Our study offers a rare view of the clinically relevant biological processes that characterize RD, which is a treatment phase that is infrequently captured in human solid malignancies.

**Figure 6:**
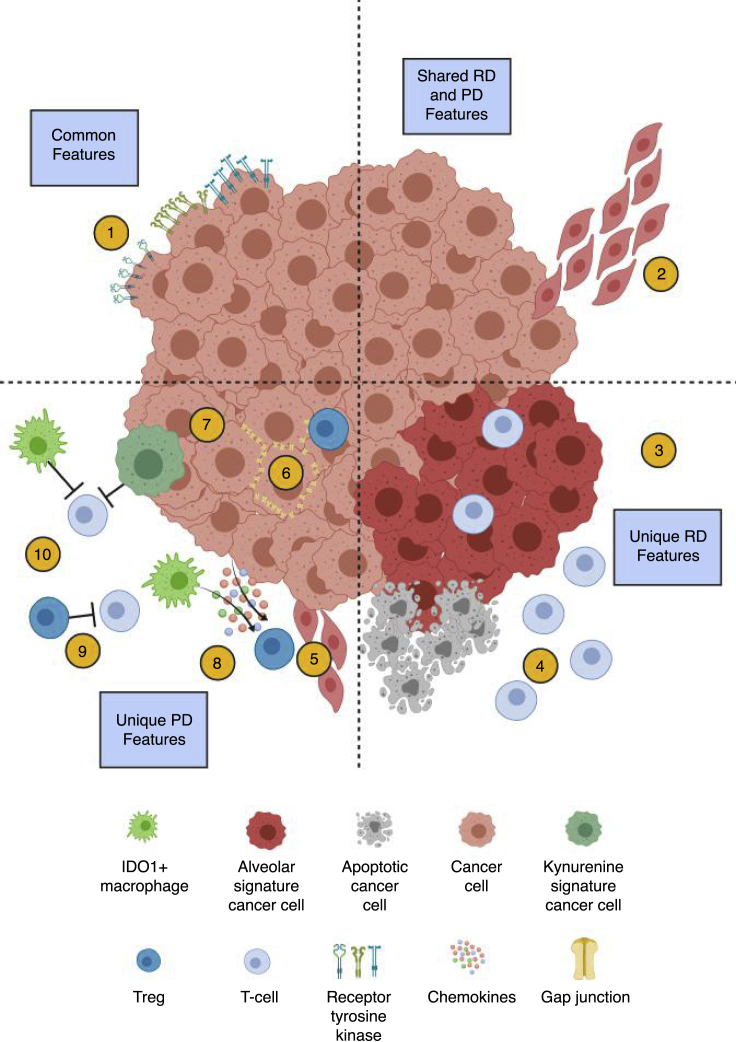
scRNA-seq Profiles Reveal Clinical-State Specific Features of the Tumor Cellular Ecosystem Features common to all time points are shown in the top-left quadrant and include the presence of multiple oncogenic drivers (1). Features shared in RD and PD are shown in the top-right quadrant and include various invasive signaling pathways (2). Features unique at RD, shown in the bottom-right quadrant, include the Alveolar signature (3) and increased T cell fraction (4). Features unique to PD, shown in the bottom-left quadrant, include upregulation of the plasminogen activation pathway (5), expression of gap-junction proteins (6), loss of tumor suppressor genes (7), expression of pro-inflammatory chemokines (8), increased Treg population (9), and increased kynurenine signature expression (10).

Our scRNA-seq data revealed widespread intra-tumoral heterogeneity in oncogenic alterations that are expressed in cancer cells (Figure 2) by demonstrating expression of not only the putative oncogenic driver but also additional oncogenic mutations (Figure 6, #1). This provides a potential explanation for why complete responses to treatment are rare. Tumors harbor the appropriate genetic framework and evolutionary playbook to evolve resistance. These “hard-wired” properties can go undetected by current bulk sampling analysis. Tumor resilience and evolution during therapy are bolstered by the therapy-induced transcriptional plasticity that we demonstrated by scRNA-seq profiling.

We uncovered transcriptional signatures specific to different treatment time points and clinical states (Figures 3 and Figure 6, #3, #5, #6, and #7). The majority of these signatures were biomarkers of significantly worse OS and were most pronounced at PD. Conversely, we found the alveolar cell signature was enriched at RD and was associated with improved survival. This signature exhibited features consistent with cellular plasticity and injury response, perhaps indicating a treatment-inducedadaptive phenotype that permits the survival of cancer cells, albeit in a less aggressive state (Wang et al., 2018). Our data also highlight a connection from the alveolar cell signature to the WNT/β-catenin pathway as a mechanism of injury response and regeneration. Though the WNT/β-catenin pathway is potentially therapeutically targetable, (Krishnamurthy and Kurzrock, 2018), it will be critical to determine how to best modulate this pathway to impact residual cancer cell survival for clinical benefit.

A general principle our data highlight is that by employing targeted treatments that take advantage of specific cell states we may be able to engineer cancer (or TME) cell fate(s) to improve therapeutic responses in metastatic solid malignancies. If deployed at the appropriate time, treatments that target liabilities of a specific cell state or prevent further adaptation may help improve patient survival by constraining continued tumor evolution toward complete drug resistance (Table 1).

**Table 1:** Table of Common and Unique Features in Different Treatment Time Points and Possible Therapeutic Approaches

ID	Feature	Therapeutic Approach	References
1	Multiple targetable mutations	Combination targeted therapy	McCoach and Bivona, 2019
2	Invasion pathways	Targeted inhibition	Rahman et al., 2017, Zhang et al., 2016, Zhang et al., 2019
3	Increased immuno-stimulatory T cells	Immune system modulation	Ha et al., 2019, Jenkins et al., 2018, Souza-Fonseca-Guimaraes et al., 2019, Valkenburg et al., 2018
4	Alveolar signature	Targeted inhibition	Nabhan et al., 2018, Zhang et al., 2019
5	Plasminogen activation signature	Targeted inhibition	Mahmood et al., 2018, Zhang et al., 2019
5	*SERPINE1* signature	Targeted inhibition, immune system modulation	Placencio and DeClerck, 2015
6	Gap-junction signature	Targeted inhibition	Mulkearns-Hubert et al., 2019, Wu and Wang, 2019
7	Loss of tumor suppressors	Targeting acquired vulnerabilities	Ding et al., 2019
8	Increased pro-inflammatory chemokines	Immune system modulation	Tokunaga et al., 2018
9	Increased tregs	Immune system modulation	Tanaka and Sakaguchi, 2019
10	Kynurenine signature	Targeted inhibition, immune system modulation	Labadie et al., 2019

The recognition of targetable oncogene-driven NSCLCs as a subset of lung cancer that is poorly responsive to current checkpoint inhibitor immunotherapies (Gainor et al., 2016; Mazieres et al., 2019) necessitates an improved understanding of the immunologic milieu in this patient population. We found a relatively low T cell infiltration in the TME of TN and PD patients (Figures 5C and 5D), consistent with prior reports of low cytotoxic T cell infiltration in treatment-naive *EGFR* mutant NSCLCs (Gainor et al., 2016) and with an association between *EGFR* activation and an immunosuppressive phenotype in preclinical models (Akbay et al., 2013; Jiang et al., 2019). Our results uncovered an induction of a more inflammatory phenotype during RD on targeted therapy, hallmarked by infiltration of T cells (Figure 6, #4) and decreased infiltration of immunosuppressive macrophages (Figure 5E). This inflammatory state may represent a complement to the alveolar cell, injury repair, and regenerative state present in the cancer cell compartment (described above), with the potential for crosstalk between the cancer cells and TME. These TME changes were transient, as at PD there was enrichment for *IDO1*-expressing macrophages, regulatory T cells, and other immunosuppressive T cell populations. These are all features of an environment hostile to the establishment of an effective immunologic response (Figure 6, #9 and #10). These findings confirm those reported within preclinical models of *EGFR* mutant NSCLCs, which demonstrated the potential for a transient immunostimulatory effect after initial EGFR TKI exposure despite the immunosuppressive phenotype observed following longer-term TKI treatment (Dominguez et al., 2016). The induction of a more immunostimulatory phenotype during targeted therapy (i.e., in RD) may offer a window-of-opportunity to introduce novel TME target-based combination therapies earlier during treatment, perhaps around the time of RD in the context of a more favorable TME to increase initial response and consolidate the anti-tumor responsein a multi-modal therapeutic approach.

Given that cancer cell signaling and the TME are linked, there may be treatment strategies that target both compartments concurrently. The kynurenine pathway is one example. We identified increased kynurenine pathway activation in cancer cells and myeloid cells at PD (Figure 6, #5). *IDO1*, as a rate-limiting enzyme in the kynurenine pathway, can influence diverse components of the TME including T cell and myeloid cell populations as well as angiogenesis in favor of immunosuppression (Munn and Mellor, 2016). The use of IDO1 inhibitors as part of a combination immunotherapy strategy with PD1/PDL1 (programmed cell death protein 1/programmed death-ligand 1) checkpoint inhibitors showed promise in early-phase studies (Siefker-Radtke et al., 2018),yet failed to demonstrate improved outcomes in advanced-stage melanoma (Long et al., 2018). We demonstrated distinct evolving TME states, suggesting that there may be a window-of-opportunity at which point kynurenine pathway inhibitors may be more effective (Figure 6; Table 1).

The scRNA-seq dataset presented here demonstrates the feasibility of performing scRNA-seq on tumor biopsies obtained longitudinally at clinically relevant time points during active targeted treatment of advanced-stage solid malignancy patients. The data provide a foundation to develop strategies for the elimination or neutralization of RD to induce more durable responses for patients with advanced-stage NSCLCs and potentially other solid malignancies across different therapeutic modalities.

### Limitation of Study

Limitations of our study include the number and diversity of cells and genotypes of individual tumor biopsies due to the use of small needle biopsies or fluid collections versus larger surgical resections. Due to the real-world challenges of tissue acquisition, we acquired matched samples from a small number of individual patients. Because RD is not sampled during standard treatment, there were fewer samples at this disease state. Single-cell-derived transcriptomes are relatively sparse as a consequence of a combination of factors including transcriptional stochasticity, rarity of sampling mRNA molecules, uneven amplification of mRNA molecules during cDNA synthesis and library preparation, and sparse read coverage of library molecules (Borel et al., 2015; Deng et al., 2014; Fan et al., 2018). These challenges limit our ability to perform a saturating mutation analysis using single-cell data.

## STAR★Methods

### Key Resources Table

**Table:** 

REAGENT or RESOURCE	SOURCE	IDENTIFIER
**Antibodies**		

CD45-FITC	Miltenyi Biotec	Cat# 1300-080-202; RRID: AB_244234
CTNNB1 rabbit monoclonal	Cell Signaling Technology	Cat# 8480S; RRID: AB_11127855
AQP4 rabbit monoclonal	Cell Signaling Technology	Cat# 59678; RRID: AB_2799571
SUSD2 rabbit polyclonal	Sigma Aldrich	Cat# HPA004117; RRID: AB_1857674
PD-L1	Cell Signaling Technologies	Clone E1L3N; RRID: AB_2687655
CD68	Dako	Clone KP1; RRID: AB_578703
IDO	Cell Signaling Technologies	Clone D5J4E; RRID: AB_2636818
HLA-DR	Abcam	Clone CR3/43; RRID: AB_443647
CD14	Abcam	Clone SP192;
Cytokeratin	Dako	Polyclonal Z0622; RRID: AB_2650434
CD3	Leica	Clone LN10; RRID: AB_10554454
PD-1	Abcam	Clone NAT105; RRID: AB_881954
CD8	Dako	Clone C8/144B; RRID: AB_2075537
FoxP3	Abcam	Clone 236A/E7; RRID: AB_445284

**Biological Samples**		

Thoracentesis, resection, tumor adjacent tumor and core biopsy samples	University of California San Francisco	N/A

**Chemicals, Peptides, and Recombinant Proteins**		

DMEM	GE Life Sciences	Cat# SH30081.01
Collagenase Type 2	Worthington Biochemical	Cat# LS004176
RBC lysis buffer	Thermo Fisher Scientific	Cat# A1049201
FBS	Omega Scientific, Inc.	Cat# FB-11
Running buffer	Miltenyi Biotec	Cat# 130-091-221
BSA	Miltenyi Biotec	Cat# 130-091-221
Hoechst stain	Thermo Fisher Scientific	Cat# H3570
PI	Life Technologies	Cat# P3566
Sytox Blue	Thermo Fisher Scientific	Cat# S34867
Recombinant RNase Inhibitor	Takara Bio	Cat# 2313B
Triton™ X-100	Sigma	Cat# 93443
dNTP mix	Thermo Fisher	Cat# R0193
ERCC RNA spike-in mix	Thermo Fisher	Cat# 4456740
SMARTScribe Reverse Transcriptase	Takara Bio	Cat# 639538
First-Strand Buffer	Takara Bio	Cat# 639538
DTT	Bioworld	Cat# 40420001-1
Betaine	Sigma	Cat# B0300
MgCl_2_	Sigma	Cat# M1028
KAPA HiFi HotStart ReadyMix	Kapa Biosystems	Cat# KK2602
Lambda Exonuclease	NEB	Cat# M0262L
Tris-HCl	Thermo Fisher	Cat# 15568025
qPCR	Kapa Biosystems	Cat# KK4923
Osimertinib	Selleck Chemicals	Cat# S7297
Alectinib	Selleck Chemicals	Cat# S2762
XAV-939	Selleck Chemicals	Cat# S1180
PRI-724	Selleck Chemicals	Cat# S8262
Sodium Bicarbonate	Millipore Sigma	Cat# S5761
Bovine Serum Albumin	VWR International	Cat# 97061-422
Tween-20	Millipore Sigma	Cat# P9416
Sodium Azide	Millipore Sigma	Cat# S2002
Xylenes	Fisher Chemical	Cat# X5-4
Hematoxylin solution	VWR International	Cat# 95057-844
Target Retrieval solution, Citrate	Agilent Dako	Cat# S169984-2
Richard-Allan Scientific Cytoseal 60	Thermo Fisher Scientific	Cat# 8310-16
Epitope Retrieval Solution 1	Lecia	Cat# AR9961
Epitope Retrieval Solution 2	Lecia	Cat# AR9640
Antibody Diluent	Akoya Biosciences	Cat# ARD1001EA
Opal Polymer HRP Ms + Rb	Akoya Biosciences	Cat# ARH1001EA
BOND Wash Solution	Lecia	Cat# AR9590
DAPI	Akoya Biosciences	Cat# FP1490
ProLong Diamond Antifade Mountant	Thermo Fisher Scientific	Cat# P36961

**Critical Commercial Assays**		

Nextera XT Library Sample Preparation kit	Illumina	Cat# FC-131-1096
NextSeq 500/550 Hi Output Kit v2.5 (300 cycle)	Illumina	Cat# 20024908
Novaseq S2 (300 cycle)	Illumina	Cat# 20012860
RT2 Profiler PCR array	QIAGEN	Cat# CLAH34795
Fragment analyzer kit	Agilent	Cat# DNF-474-0500
Tapestation D5000 Kit	Agilent	Cat# 5067-5593
Tapestation D5000 Tapes	Agilent	Cat# 5067-5592
EnVision+ Dual Link Kit	Agilent Dako	Cat# K406511-2
Opal IHC Multiplex Assay	Perkin Elmer	Cat# NEL801001KT

**Deposited Data**		

MSK-Impact	(Cerami et al., 2012; Gao et al., 2013)	https://www.mskcc.org/msk-impact
TCGA	TCGA Research Network; (Liu et al., 2018)	https://www.cancer.gov/about-nci/organization/ccg/research/structural-genomics/tcga
dbSNP	(Sherry et al., 2001)	https://www.ncbi.nlm.nih.gov/variation/docs/human_variation_vcf/
COSMIC (Catalogue of Somatic Mutations in Cancer)	(Tate et al., 2019)	https://cancer.sanger.ac.uk/cosmic/download
Normal AT2 single-cell gene counts	(Vieira Braga et al., 2019)	GEO-GSE130148
scRNaseq NSCLC	This study	BioProject- PRJNA591860

**Experimental Models: Cell Lines**		

PC9 cells	ATCC	N/A
H3122 cells	ATCC	N/A

**Oligonucleotides**		

Oligo-dT_30_VN-5′AAGCAGTGGT ATCAACGCAGAGTACT_30_VN-3′	IDT	N/A
TSO-5′AAGCAGTGGTA TCAACGCAGACTACATrGrG+G-3′	Exiqon	N/A
IS PCR primer-5′AAGCAGTGGTATCAACGCAGAGT-3′	IDT	N/A

**Software and Algorithms**		

R	(R, 2013)	https://www.r-project.org/
bcl2fastq	Illumina	https://support.illumina.com/sequencing/sequencing_software/bcl2fastq-conversion-software.html; RRID:SCR_015058
STAR	(Dobin et al., 2013)	https://github.com/alexdobin/STAR
HTSEQ	(Anders et al., 2015)	https://htseq.readthedocs.io/en/master/
Rstudio	(RStudio 2015)	https://rstudio.com/
Seurat v3.0	(Stuart et al., 2019)	https://github.com/satijalab/seurat
DoubletFinder	(McGinnis et al., 2019)	https://github.com/chris-mcginnis-ucsf/DoubletFinder
inferCNV	(Tickle et al., 2019)	https://github.com/broadinstitute/inferCNV.).
MAST	(Finak et al., 2015)	https://github.com/RGLab/MAST
pheatmap	(Kolde, 2019)	https://cran.r-project.org/web/packages/pheatmap/index.html
dyplr	(Wickham et al., 2020)	https://cran.r-project.org/web/packages/dplyr/index.html
ggplot2	(Wickham, 2016)	https://cran.r-project.org/web/packages/ggplot2/index.html
Reflow	GRAIL	https://github.com/grailbio/reflow
survival	(Therneau, 2015)	https://cran.r-project.org/web/packages/survival/index.html
survminer	(Kassambara et al., 2019)	http://cran.r-project.org/web/packages/surviminer/index.html
GATK HaplotypeCaller	(DePristo et al., 2011)	broadinstitute/gatk:4.0.11.0
fathmm	(Shihab et al., 2015)	https://github.com/HAShihab/fathmm
STAR-fusion	(Haas et al., 2019)	https://github.com/STAR-Fusion/STAR-Fusion/wiki,
cerebra	(Unpublished data)	https://pypi.org/project/cerebra/
lifelines	(Davidson-Pilon et al., 2019)	https://github.com/CamDavidsonPilon/lifelines/
Python v3.4	(Python, 2015)	https://python.org/
REdaS	(Maier, 2015)	N/A
Phenochart v1.0.8	Perkin Elmer	N/A
inForm v2.4.8	Akoya	N/A
CellInsight	Thermo Fisher Scientific	N/A
BioRender	BioRender	N/A
Adobe Illustrator	Adobe	N/A

**Other**		

Mosquito 384w Spool of 4.5mm tips	TTP Labtech	Cat# 4150-03010
Mantis Low Volume Chip	Fisher Scientific	Cat# NC1491372
Mantis High Volume Chip	Fisher Scientific	Cat# NC1491373
RNeasy Mini Kit	QIAGEN	Cat# 74104
Bioanalyzer RNA 6000 Pico kit	Agilent	Cat# 5067-1514
Qubit RNA HS Assay kit	Thermo Fisher Scientific	Cat# Q32852
First Strand Synthesis Kit	QIAGEN	Cat# 330401
AMPure beads	Fisher	Cat# A63881
100-micron filter	Fisherbrand	Cat# 22363548
FACS tube	Falcon	Cat# 14-956-3C
384-well hard-shell PCR plates	BioRad	Cat# HSP3901
Fisherfinest Premium Cover Glasses (50 × 24 mm)	Fisher Scientific	Cat# 12-548-5M

### Resource Availability

#### Lead Contact

Further information and requests for resources should be directed to and will be fulfilled by the Lead Contact, Trever Bivona (https://cran.r-project.org/web/packages/REdaS/index.html).

#### Materials Availability

This study did not generate new unique reagents.

#### Data and Code Availability

The dataset generated during this study is available as an NCBI BioProject #PRJNA591860. All code used to generate the results of this study can be found on github at czbiohub/scell_lung_adenocarcinoma and czbiohub/cerebra. The below methods reference specific code notebooks (script xx) available at czbiohub/scell_lung_adenocarcinoma to analyze data.

### Experimental Model and Subject Details

#### Human Subjects

All patients gave informed consent for collection of clinical correlates, tissue collection, research testing under Institutional Review Board (IRB)-approved protocols (CC13-6512 and CC17-658, NCT03433469). Patient demographics are listed in Table S1. Patient studies were conducted according to the Declaration of Helsinki, the Belmont Report, and the U.S. Common Rule.

#### Cell Lines

PC9 (EGFR^exon19del^) and H3122 (EML4-ALK^v1^) cells were purchased from ATCC and grown in a 5% CO_2,_ humidified atmosphere at 37°C. Cultures were maintained using RPMI 1640 medium (GE Healthcare) supplemented with 10% (v/v %) fetal bovine serum (VWR), 100 IU/mL penicillin and 100 μg/mL streptomycin (GIBCO).

### Method Details

#### Patient population

Formalin-fixed paraffin embedded (FFPE), frozen, and fresh tissue samples were obtained according to the safety standards of the interventional radiologist, pulmonologist, or surgeon. Demographic and clinical history for each patient was obtained from chart review and is listed in Table S1. Days until progression were determined based on imaging studies which demonstrated definitive growth of a known tumor site or new extra-CNS metastatic deposits. Residual disease state was determined by serial imaging demonstrating continued reduction or stability tumor with no evidence of progression. Complete details of each patient sample acquisition are outlined in Table S1 and Figure S1A. Additionally, the timing of each sample acquisition is shown in Figure S1A.

#### Sample preparation of cores and resections

Tissue was first cut into small pieces and placed into a 1.5 mL tube (or multiple tubes if necessary). 1.5 mL of collagenase buffer (10mL DMEM (GE Life Sciences, SH30081.01), 0.20 g Collagenase Type 2 (Worthington Biochemical, LS004176)) was added to the tube and the sample was digested for 30 minutes at 37°C, shaking in a thermomixer @ 800-1000 rpm. The sample was manually agitated by pipetting up and down 15 times then returned to the thermomixer for 25 minutes. After incubation, the sample was removed from the thermomixer, agitated again by pipetting the sample up and down 15 times before passing the sample through a 100-micron filter (Fisherbrand, 22363548) into a new 15 mL falcon tube. The filter was washed with 1-2 mL of collagenase buffer before the sample was spun in the centrifuge at 500xg for 10 minutes. If the resulting cell pellet was red, 0.5 mL RBC lysis buffer (Thermo Fisher Scientific, A1049201) was added to sample tubes and allowed to sit at room temperature for 3 minutes before quenching with 1.0 mL DMEM (GE Life Sciences, SH30081.01) + 6% FBS (Omega Scientific, Inc, FB-11) and spun in the centrifuge at 500xg for 5 minutes. Remaining cells were stained with 10 μl CD45-FITC (Miltenyi Biotec, 130-080-202) and 1 μl of Hoechst stain (Thermo Fisher Scientific, H3570). Samples incubated on ice in the dark for 20 minutes. 1mL of FACS Buffer (1:20 dilution of BSA (Miltenyi Biotec, 130-091-221) in Running Buffer (Miltenyi Biotec, 130-091-221)) was then added to the stained cells and spun at 500xg for 10 minutes before aspirating off supernatant. Cells were resuspended with 0.5 mL of FACS Buffer. PI (Life Technologies, P3566) was added immediately prior to sorting.

#### Sample preparation of thoracentesis samples

Cells were filtered through a 100 μm strainer (Fisherbrand 22363548), pelleted (500xg, 5 min, 4°C), and resuspended in FACS buffer. Cells were then stained with CD45-FITC (Miltenyi Biotec, 130-080-202) for 20 min at 4°C in the dark. Cells were then pelleted (500xg, 5 min, 4°C) and resuspended in FACS buffer before being transferred to a FACS tube (Falcon 14-956-3C). Sytox Blue dead cell stain (Thermo Fisher Scientific, S34867) was added immediately prior to sorting.

#### Lysis plate preparation

Lysis plates were created by dispensing 0.4 μL lysis buffer (0.5U Recombinant RNase Inhibitor (Takara Bio, 2313B), 0.0625% Triton™ X-100 (Sigma, 93443-100ML), 3.125 mM dNTP mix (Thermo Fisher, R0193), 3.125 μM Oligo-dT_30_VN (IDT, 5′AAGCAGTGGTATCAACGCAGAGTACT_30_VN-3′) and 1:600,000 ERCC RNA spike-in mix (Thermo Fisher, 4456740)) into 384-well hard-shell PCR plates (Biorad HSP3901) using a Tempest liquid handler (Formulatrix). All plates were then spun down for 1 minute at 3220xg and snap frozen on dry ice. Plates were stored at −80°C until used for sorting.

#### FACS sorting

Cells were sorted into 384-well plates using SH800S (Sony) sorter. Cells were sorted using the “Ultra purity” setting on the sorter. For a typical sort, a FACs tube containing 0.3-1ml the pre-stained cell suspension was vortexed gently and loaded onto the FACS machine. A small number of cells were flowed at low pressure to check cell concentration and amount of debris. Then the pressure was adjusted, flow was paused, the first destination plate was unsealed and loaded. Single-cell sorting was done where half the plate was sorted for CD45+/PI-/Hoechst+ while the second half was sorted for CD45-/PI-/Hoechst+. Immediately after sorting, plates were sealed with a pre-labeled aluminum seal, centrifuged and flash frozen on dry ice.

#### cDNA synthesis and library preparation

cDNA synthesis was performed using the Smart-seq2 (Picelli et al., 2013; Picelli et al., 2014; Tabula Muris et al., 2018). Briefly, 384-well plates containing single-cell lysates were thawed on ice followed by first strand synthesis. 0.6 μL of reaction mix (16.7 U/μl SMARTScribe Reverse Transcriptase (Takara Bio, 639538), 1.67 U/μl Recombinant RNase Inhibitor (Takara Bio, 2313B), 1.67X First-Strand Buffer (Takara Bio, 639538), 1.67 μM TSO (Exiqon, 5′-AAGCAGTGGTATCAACGCAGACTACATrGrG+G-3′), 8.33 mM DTT (Bioworld, 40420001-1), 1.67 M Betaine (Sigma, B0300-5VL), and 10 mM MgCl_2_ (Sigma, M1028-10X1ML)) was added to each well using a Tempest liquid handler or Mosquito (TTP Labtech). Reverse transcription was carried out by incubating wells on a ProFlex 2x384 thermal-cycler (Thermo Fisher) at 42°C for 90 min and stopped by heating at 70°C for 5 min.

Subsequently, 1.5 μL of PCR mix (1.67X KAPA HiFi HotStart ReadyMix (Kapa Biosystems, KK2602), 0.17 μM IS PCR primer (IDT, 5′-AAGCAGTGGTATCAACGCAGAGT-3′), and 0.038 U/μl Lambda Exonuclease (NEB, M0262L)) was added to each well with a Mantis liquid handler (Formulatrix) or Mosquito, and second strand synthesis was performed on a ProFlex 2x384 thermal-cycler by using the following program: 1. 37°C for 30 minutes, 2. 95°C for 3 minutes, 3. 23 cycles of 98°C for 20 s, 67°C for 15 s, and 72°C for 4 minutes, and 4. 72°C for 5 minutes.

The amplified product was diluted with a ratio of 1-part cDNA to 10 parts 10mM Tris-HCl (Thermo Fisher, 15568025). 0.6 μL of diluted product was transferred to a new 384-well plate using the Viaflow 384 channel pipette (Integra). Illumina sequencing libraries were prepared as described in Darmanis et al. (2015). Briefly, tagmentation was carried out on double-stranded cDNA using the Nextera XT Library Sample Preparation kit (Illumina, FC-131-1096). Each well was mixed with 0.8 μL Nextera tagmentation DNA buffer (Illumina) and 0.4 μL Tn5 enzyme (Illumina), then incubated at 55°C for 10 min. The reaction was stopped by adding 0.4 μL “Neutralize Tagment Buffer” (Illumina) and spinning at room temperature in a centrifuge at 3220xg for 5 min. Indexing PCR reactions were performed by adding 0.4 μL of 5 μM i5 indexing primer, 0.4 μL of 5 μM i7 indexing primer, and 1.2 μL of Nextera NPM mix (Illumina). All reagents were dispensed with the Mantis or Mosquito liquid handlers. PCR amplification was carried out on a ProFlex 2x384 thermal cycler using the following program: 1. 72°C for 3 minutes, 2. 95°C for 30 s, 3. 12 cycles of 95°C for 10 s, 55°C for 30 s, and 72°C for 1 minute, and 4. 72°C for 5 minutes.

#### Library sequencing

Following library preparation, wells of each library plate were pooled using a Mosquito liquid handler. Pooling was followed by two purifications using 0.7x AMPure beads (Fisher, A63881). Library quality was assessed using high sensitivity capillary electrophoresis on a Fragment Analyzer (Agilent) or Tapestation (Agilent), and libraries were quantified by qPCR (Kapa Biosystems, KK4923) on a CFX96 Touch Real-Time PCR Detection System (Biorad). Plate pools were normalized to 2 nM and equal volumes from library plates were mixed together to make the sequencing sample pool.

#### Sequencing libraries from 384-well plates

Libraries were sequenced on the NextSeq or NovaSeq 6000 Sequencing System (Illumina) using 2 × 100bp paired-end reads and 2 × 8bp or 2 × 12bp index reads. NextSeq runs used high output kits, whereas NovaSeq runs used either a 200 or 300-cycle kit (Illumina, 20012860). PhiX control library was spiked in at ∼1%.

#### Immunohistochemistry

All specimens were acquired from individuals with NSCLC as noted above. 4-micron thick formalin-fixed paraffin embedded (FFPE) human tissue sections were processed using previously published method (Haderk et al., 2019) and Agilent-Dako manufacturer recommendations were followed for antigen retrieval. All wash steps were performed at room temperature for three minutes each, unless otherwise noted. Briefly, slides were deparaffinized in xylenes (2 washes, 5 min each), and rehydrated in graded dilutions of aqueous ethanol (2 washes in 100% EtOH; 2 washes in 95% EtOH; 1 wash in 70% EtOH). Slides were washed once in ddH2O before being placed in an antigen target retrieval solution, 1x pH 6.1 Citrate retrieval solution (Dako) and pressure cooked using one cycle (2 hours) for antigen retrieval. Slides were allowed to cool to room temperature, washed three times with 1x PBS, then the tissue was blocked for endogenous peroxidase activity for 10 minutes using 0.3% H2O2. Slides were washed three times with 1x PBS, then incubated for 1 hour in a prepared protein blocking buffer solution (1X PBS containing 1% (w/v) BSA, 15 mM sodium azide, 0.05% (w/v) Tween-20). Slides were incubated overnight at 4C with either β-catenin (CTNNB1) rabbit monoclonal antibody (#8480S, Cell Signaling Technology, 1:100 dilution), AQP4 rabbit monoclonal antibody (#59678, Cell Signaling Technology, 1:100 dilution), or SUSD2 rabbit polyclonal antibody (HPA004117, Sigma Aldrich, 1:400 dilution). The following morning, the slides were washed three times with 1x PBS, incubated using commercial anti-rabbit and anti-mouse labeled polymer-HRP solution (Agilent Dako) for 30 minutes. Slides were washed three times in 1x PBS before incubation with freshly prepared 3,3-diaminobenzidine chromogen solution (Agilent Dako) for < 1 minute. Slides were washed twice in ddH2O and were counterstained using a commercial hematoxylin solution (VWR Biosciences). Excess dye was removed using three washes in ddH2O, and the hematoxylin was developed by incubating for 1 minute in 0.1% (w/v) sodium bicarbonate solution, and washed once in ddH2O. Tissues were dehydrated in aqueous ethanol (2 washes in 95% EtOH; 2 washes in 100% EtOH) and incubated in xylene for 5 minutes before being coverslipped. Stained slides were digitized using an Aperio ScanScope XT Slide Scanner (Leica Biosystems) using a 40X objective.

#### Multiplex Immunofluorescence

Multiplex immunofluorescence staining was performed on the Opal IHC Multiplex Assay (NEL821001KT, Akoya Biosciences). Sequential 4 micron sections mounted on glass slides were sequentially stained for panel 1: PD-L1 (clone E1L3N, dilution 1:50, Cell Signaling Technologies), CD68 (clone KP1, dilution 1:500, Dako), IDO (clone D5J4E, dilution 1:100, Cell Signaling Technologies), HLA-DR (clone CR3/43, dilution 1:250, Abcam), CD14 (clone SP192, dilution 1:100, Abcam), and cytokeratin (polyclonal Z0622, dilution 1:250, Dako); or panel 2: CD3 (clone LN10, Leica), PD-1 (clone NAT105, dilution 1:100, Abcam), CD14, CD8 (clone C8/144B, dilution 1:100, Dako), FoxP3 (clone 236A/E7, dilution 1:200, Abcam), and cytokeratin on a Bond RX autostainer (Leica Biosystems). Slides were dewaxed (Leica), heat treated in Epitope retrieval solution 1 or 2 (AR9961/AR9640, Lecia) buffer depending on the antibody for 20 min at 93C, blocked in Antibody (Ab) Diluent (ARD1001EA, Akoya Biosciences), incubated for 30 min with the primary Ab, 10 min with horseradish peroxidase (HRP)-conjugated secondary polymer (anti-rabbit and anti-mouse, ARH1001EA, Akoya Biosciences), and 10 min with HRP-reactive OPAL fluorescent reagents (NEL821001KT, Akoya Biosciences). Slides were washed between staining steps with Bond Wash (AR9590, Leica) and stripped between each round of staining with heat treatment in antigen retrieval buffer. After the final heat treatment in antigen retrieval buffer, the slides were stained with spectral DAPI (FP1490, Akoya Biosciences), and coverslipped with Prolong Diamond mounting media (P36961, Thermo Fisher). Whole slide scans were acquired using the 10x objective via the Vectra imaging system (Perkin Elmer, version 3.0).

#### RT PCR *in vitro* system gene expression

For validation of candidate gene expression via a RT2 Profiler PCR array (QIAGEN, CLAH34795), human lung cancer PC9 cells (5 × 10^**5**^) were treated for 48 hours (day 2) with DMSO (TN) or for 7 and 19 days with 2μM Osimertinib (Selleck Chemicals, S7297) with replenishment of drug every 3-4 days (Persister cells that evade drug-induced apoptosis by being in a low- to no-proliferative state, in patients this corresponds to the RD state), respectively. PD samples were derived from an acquired resistant PC9 cell line (Osimertnib IC_50_ = 89μM), that was generated by continuous treatment with 2μM Osimertinib with replenishment of drug every 3-4 days and presented *active proliferation* under drug at which time they were considered to be resistant and in the PD state. RNA was extracted via RNeasy Mini Kit (QIAGEN, 74104). RNA quality was confirmed as RIN > 7.5 via Bioanalyzer RNA 6000 Pico kit (Agilent, 5067-1514) and RNA was quantified via Qubit RNA HS Assay kit (Thermo Fisher Scientific, Q32852). A total of 400ng of RNA was reverse transcribed using the First Strand Synthesis Kit (QIAGEN, 330401) and then loaded into a custom 384 well RT2 profiler array (QIAGEN, CLAH34795).

#### Wnt/β-catenin inhibition

Small molecule inhibitors were all purchased commercially from Selleck Chemicals, and included Osimertinib (S7297), Alectinib (S2762), XAV-939 (S1180), and PRI-724 (S8262). Dimethyl sulfoxide (DMSO) (Fisher Scientific) was used to dissolve small molecule inhibitors according to manufacturer’s recommendations for use in *in vitro* experiments. PC9 and H3122 cells (5 × 10^3^) were seeded in 96-well plate format (μclear CellStar, Greiner) and rested for 24 hours before treatment. Treatment included: i) DMSO, ii) tyrosine kinase inhibitors (TKI) Osimertinib (PC9 cells) or Alectinib (H3122 cells), iii) Wnt/β-catenin inhibitors PRI-724 or XAV-939, and v) indicated combination therapies of TKI and Wnt/ β-catenin inhibitors. All conditions were plated in technical quadruplicate and cells were retreated every 3 days. At each imaging interval, cellular nuclei were stained with Hoechst 33342 (Thermo Fisher Scientific) and scanned using a CellInsight High-Content Microscope (Version 6.4.3 Build 7204, Thermo Fisher Scientific) with a 4X objective.

### Quantification and Statistical Analysis

#### Alignment and gene counts

Sequences from the Illumina sequencing were demultiplexed using bcl2fastq version 2.19.0.316 (Illumina). Reads were aligned using the hg38 genome using STAR version 2.5.2b (Dobin et al., 2013) with parameters TK. Gene counts were produced using HTSEQ version 0.6.1p1 (Anders et al., 2015) with default parameters except stranded was set to false and mode was set to intersection-nonempty.

#### General clustering

Standard procedures for filtering, variable gene selection, dimensionality reduction, and clustering were performed using the Seurat v3 (Stuart et al., 2019) in RStudio (RStudio, 2015) using R (R, 2013), where cells with fewer than 500 genes and 50,000 reads were excluded. We used DoubletFinder (McGinnis et al., 2019) to identify potentially sorted doublet cells. 218 doublets were excluded from further analysis. Samples with less than 10 total cells were filtered from the analysis. Counts were log-normalized, then scaled by linear regression against the number of reads. Variable genes (Ngenes = 2,000) were selected using a threshold for dispersion, with z-scores normalized by expression level. The variable genes were projected onto a low-dimensional subspace using principal component analysis. The number of principal components (Npcs) were selected based on inspection of the plot of variance explained (Npcs = 20). A shared-nearest-neighbors graph was constructed based with metric the Euclidean distance in the low-dimensional subspace. Cells were visualized using a 2-dimensional tSNE on the same distance metric (Res = 0.5, Kparam = 30, script 03). Cell types were assigned to each cluster of cells using the abundance of known marker genes (Table S2, script S01-03 and script NI01).

#### Epithelial subset analysis

Cells previously annotated as epithelial (n = 5,581) were subset and re-clustered using methods described above and the following parameters: Ngenes = (2,000), Npcs = 20, Res = 0.7, Kparam = 30 (script NI02). Malignant epithelial cells were identified using inferCNV (Tickle et al., 2019). inferCNV which works by finding cells with large copy number variations as determined by sorting expressed genes by their chromosomal location and applying a moving average, a sliding window of 100 genes within each chromosome, to the relative expression values (Patel et al., 2014; Puram et al., 2017; Tirosh et al., 2016). All epithelial cells as well as 300 fibroblasts and 300 endothelial cells were used as input (script NI03). An additional 500 fibroblasts and 500 endothelial cells were used as reference controls. We scored each cell for the extent of CNV signal and plotted cells on a dendrogram which was then cut at the highest point in which all the spiked in endothelial and fibroblasts cells belonged to one cluster (k = 6, one fibroblast control was misassigned). All cells that clustered together with spiked in controls were labeled “nontumor,” whereas the remaining two clusters were labeled as “tumor.”

Noncancerous epithelial cells (n = 1,827), as determined as those cells lacking large chromosomal aberrations from InferCNV analysis, were subset and re-clustered using the following parameters: Ngenes = (2,000), Npcs = 20, Res = 0.5, Kparam = 20 (script NI05). Cell types were assigned to each cluster of cells using the abundance of known marker genes (Table S2) and differentially expressed genes as found by using the Seurat function *FindAllMarkers* using the default Wilcoxon rank sum test.

#### Cancer cell subset analysis

Cancerous epithelial cells (n = 3,754), as determined as those cells harboring large chromosomal aberrations from InferCNV analysis, were subset and re-clustered using the following parameters: Ngenes = 2,000, Npcs = 20, Res = 0.9, Kparam = 10 (script NI04). We found the differences in gene expression between the three treatment time points (TN, RD, and PD) using the Seurat function *FindMarkers* using the MAST test (Finak et al., 2015) and sample_name as the latent variable. Three separate tests were used to ascertain the differences between: 1) TN and RD, 2) TN and PD and 3) RD and PD (Table S5). Resulting differential gene lists were then filtered to limit patient specific effects. This is achieved by setting a threshold for non-zero expressing cells per patient (RD = 3 of RD patients and PD = 6 of PD patients) and removing differentially expressed genes explained by less than the thresholds set. The top 100 genes from each comparison were manually curated to evaluate for pathway activation. Decreased expression could indicate lack of detection due to the stochasticity of scRNaseq and thus for analysis of activated pathways we focused on upregulated genes. Gene signatures (Table S2) were compiled using differential expressed as well as known cell marker genes. Specifically, the alveolar signature is made of differentially expressed AT1/AT2 genes among the cancer cell time point comparisons as well has additional known AT1/AT2 genes (Vieira Braga et al., 2019; Wade et al., 2006). The remaining signatures were identified directly from top differentially expressed genes.

To ensure that we were not misclassifying healthy AT2 cells as cancer cells, we compared the expression levels of our combined alveolar gene signature between the three time points (TN, RD, PD) and non-cancer AT2 cells from our dataset as well as additional non-cancer AT2 cells from an external dataset (Vieira Braga et al., 2019). Non-cancer AT2 cells from our dataset were more similar to the external AT2 cells than any of our cancer cells across all time points (average spearman correlation coefficient = 0.65, −0.10, 0.24, −0.19, for non-cancer AT2 cells, and TN, RD, PD cancer cells respectively).

Cancer cells from EGFR and ALK driven tumor samples were subset separately. We compared all three treatment time points (TN, RD, PD) for EGFR patients where we only compared two treatment time points for ALK (TN, PD) as only one ALK+ driven sample represented the RD time point. We then compared the five cancer cell signatures derived from the grouped analysis (alveolar, kynurenine, plasminogen activation, *SERPINE1*, and gap junction). Pairwise wilcoxon tests were calculated between each treatment time point (TN, RD, and PD).

To understand the PD sample heterogeneity all cancer cells from PD samples were subset. Each sample’s average expression of genes included in gene signatures (alveolar, kynurenine, plasminogen activation, *SERPINE1*, and gap junction, Figures S3B and S3F–S3H) and overall signature score was calculated and plotted using the R pheatmap package (Kolde, 2019)

Longitudinal analysis of a single patient was done by subsetting all cells originating from patient TH226. As above, the differences in gene expression between the three treatment time points (TN, RD, and PD) was found by applying the Seurat function *FindMarkers* using the MAST test (Finak et al., 2015) with sample_name as the latent variable. Three separate tests were used to ascertain the differences between: 1) TN and RD, 2) TN and PD and 3) RD and PD (script NI07-08, Table S4)

#### Survival analysis of cancer gene signatures

TCGA LUAD data were downloaded from https://xenabrowser.net/datapages/. Metadata was downloaded from *An Integrated TCGA Pan-Cancer Clinical Data Resource* Liu et al., 2018. Mean expression of each cancer cell expression signature (alveolar, kynurenine, plasminogen activating, *SERPINE1*, and gap junction) was calculated per TCGA sample. TCGA samples were then split by quartile groups. Quartiles were plotted using library packages survival (Therneau, 2015) and survminer (Haas et al., 2019) in R (script NI10). Log rank p values are reported for each signature across four expression quartiles. Cox hazard regression model was computed for comparison of quartile 1 (low expressors) versus quartile 4 (high expressors) for all signatures.

#### Analysis of immunohistochemistry

Tumor populations were annotated, then scored in a blinded, randomized analysis by a clinical pathologist for percent tumor positivity and subcellular staining intensity at the membrane, cytosolic, and nuclear compartments. SUSD2 membrane staining was graded by two reviewers in a blinded, randomized fashion using the slides annotated for tumor presence. Staining intensity was graded as negative, weak, intermediate, or strong and received scores of 0, 1, 2, or 3 respectively. Percent tumor positivity coefficient was graded as 0, negative; 1, less than 10% immunopositive; 2, between 10%–50% immunopositive; 3, between 51%–80% immunopositive; 4, greater than 80% immunopositive. Calculation of immunoreactivity scores was performed by multiplying the staining intensity score (0-3) with the percent tumor positive coefficient (0-4) to yield a value between 0 and 12 (Fedchenko and Reifenrath, 2014).

#### Mutation detection from scRNaseq

Alignment bams for all non-immune cells (stroma and epithelial) were passed to GATK HaplotypeCaller (DePristo et al., 2011) which was run from the latest available Docker container (broadinstitute/gatk:4.0.11.0) using the following options:

• –disable-read-filter MappingQualityReadFilter
• –disable-read-filter GoodCigarReadFilter
• –disable-read-filter NotSecondaryAlignmentReadFilter
• –disable-read-filter MappedReadFilter
• –disable-read-filter MappingQualityAvailableReadFilter
• –disable-read-filter NonZeroReferenceLengthAlignmentReadFilter
• –disable-read-filter NotDuplicateReadFilter
• –disable-read-filter PassesVendorQualityCheckReadFilter
• –disable-read-filter WellformedReadFilter

Disabling these specific read filters proved necessary for scRNaseq, as inherent low-coverage causes the vast majority of reads to be flagged for removal otherwise. The full human variant set (dbSNP) was downloaded from NCBI (https://www.ncbi.nlm.nih.gov/variation/docs/human_variation_vcf/), and every variant call was assessed for its presence/absence in the human variant database. dbSNP is a public, living catalog of ∼674 million human somatic SNPs and indels that have been reported by peer-reviewed publications (Sherry et al., 2001).

Cloud-based parallelization of *HaplotypeCaller* jobs was achieved with Reflow, a workflow engine for distributed, incremental data processing in the cloud (GRAIL, https://github.com/grailbio/reflow). *HaplotypeCaller* outputs a separate variant calling format file (VCF) for each cell, which were processed with the python package *cerebra* (https://github.com/czbiohub/cerebra). Variants found in dbSNP were removed, not to be included in further analysis. We reasoned that by removing ‘common’, population-level variants, we could better hone-in on disease specific variation.

In addition to scRNaseq reads, we obtained bulk DNA reads from peripheral blood for the majority of our patients (with the exception of three). These PBMC reads were run through *HaplotypeCaller* to establish ‘germline’ mutation profiles for each of our patients. Germline mutations were then subtracted out from each of that patient’s single cell VCFs. This filtering step was omitted for the three patients for which we did not obtain peripheral blood, however, these single cell VCFs were still passed through our dbSNP filter.

We also applied a *fathmm* filter to all cells (Shihab et al., 2015). *fathmm* takes a machine learning approach to predict the likelihood of a given SNP to be pathogenic, integrating ENCODE annotations for things like transcription factor binding sites, histone modifications, cross-species sequence alignment and conservation scores, etc. Only variants computationally predicted to be pathogenic were included in our analysis, i.e., those variants with a *fathmm* score > 0.7.

The remaining variants were then filtered through the COSMIC (Catalogue of Somatic Mutations in Cancer) complete mutation–genome screens database (Tate et al., 2019) (https://cancer.sanger.ac.uk/cosmic/download). Only SNPs/indels associated with ‘Lung’ as per their COSMIC annotation were kept. Variant calls were mapped to their corresponding genes, and per-patient / per-sample mutational profiles were established. We used the ERCCs spiked into each cell sample as a negative control for false positive mutations, which can arise due to technical artifacts such as PCR errors. We found the median false positive mutation rate to be 0.000256% per (Enge et al., 2017).

#### Fusion detection from scRNaseq

Fusion transcripts were detected with STAR-fusion (Haas et al., 2019) (https://github.com/STAR-Fusion/STAR-Fusion/wiki) version 1.6.0, run from a Docker container (trinityctat/ctatfusion:1.5.0). The following options were used: *–FusionInspector validate*, *–examine_coding_effect*, *–denovo_reconstruct*. Distributed processing of STAR-fusion jobs was accomplished with Reflow. Output files were processed with *cerebra*, then combined with variant calls to create per-cell and per-sample summary tables.

#### Mutational analysis of tumor cells

Mutation information from *cerebra* outputs were summarized by sample. Coverage information was provided by a secondary output from *cerebra* summarized by sample and gene. Where all cells are summarized by sample and all *fathmm* (Shihab et al., 2015) filtered ROIs are summarized by corresponding gene (script NI06). Plots were generated using the R pheatmap package (Kolde, 2019). Two comprehensive tables, Tables S3 and S7, detail mutations and fusions per cell.

#### Survival analysis within the MSK-Impact data

MSK-Impact data was downloaded from cBioPortal (Cerami et al., 2012; Gao et al., 2013) (and subset to only NSCLC samples MSK-Impact data was subset to only those mutations that were also found in the scRNaseq dataset of mutations (n = 141 unique mutations)). We stratified MSK-Impact samples by those with greater than or equal to 2 mutations from the tier one COSMIC mutations found in the scRNaseq dataset (mutation high), and those less than 2 mutations (mutation low) (Figure 2D). Kaplan-Meier plots were visualized with the lifelines package (Davidson-Pilon et al., 2019) in python version 3.4 (Python, 2015) (script NI12).

#### General immune analysis

All cells annotated as immune (n = 13,431) were subset and clustered as described above (script IM01) using the following parameters (Ngenes = 2000, Npc = 20, Res = 0.7). The resulting 18 clusters were assigned to different major immune cells types using a list of curated gene markers (Table S2) and by manual curation of differentially expressed genes for each cluster (Table S4). The different cell types and number of cells belonging to each type are described in the main text.

To assess changes in fractional abundance of different immune cell populations we used all cells though excluded thoracentesis and brain samples due to difference in the immune makeup of these tumor environments which would skew the data. The function *freqCI* from the R package REdaS (Maier, 2015) (script IM02) was used to calculate confidence intervals for relative frequencies.

Macrophages (n = 1,379) and T cells (n = 2,226) from lung biopsies were subset and clustered as described above (script IM03 and IM04 respectively) using the following parameters for MFs (Ngenes = 2000, Npc = 10, res = 0.3) and T cells (Ngenes = 2000, Npc = 10, res = 0.3). The resulting clusters are discussed in the main text and the lists of differentially expressed genes are provided (Table S4). We repeated this analysis where we subset the data to only patients with multiple biopsies and sufficient cells (TH226 and TH266) (script IM05).

#### Analysis of multiplex Immunofluorescence

Three to six regions from each slide containing tumor and stroma were selected utilizing Phenochart (v1.0.8, Perkin Elmer) for high resolution multispectral acquisition on the Vectra system at 20X magnification. The images were analyzed with inForm software (v2.4.8, Akoya) to unmix adjacent fluorochromes, subtract autofluorescence, segment the tissue into tumor and stroma regions, segment the cells into nuclear, cytoplasmic, and membrane compartments, and to phenotype the cells according to morphology and cell marker expression. Fractions of macrophage and T cell populations were calculated as: (population of interest) / (macrophage + T cell populations) and plotted using ‘ggplot2′ (Wickham, 2016) in R.

#### Immune survival analysis within the TCGA

As with the survival analysis using cancer cell gene signatures, we used the downloaded TCGA LUAD dataset and metadata to access patient survival outcomes as they pertain to the fractional changes of immune populations within a given tumor. We used CIBERSORT Newman et al., 2015 to deconvolute the bulk TCGA samples into relative fractions of immune cell populations as determined by using the LM22 reference. The total macrophage population was found by combining fractions for Monocytes, Macrophages.M0, Macrophages.M1, and Macrophages.M2. The total T cell population was found by combining fractions of T.cells.CD8, T.cells.CD4.naive, T.cells.CD4.memory.resting, T.cells.CD4.memory.activated, T.cells.follicular.helper, T.cells.regulatory.Tregs, T.cells.gamma.delta, NK.cells.resting, and NK.cells.activated. TCGA samples were then split by quartile groups. Quantiles were plotted using library packages survival (Therneau, 2015) and survminer (Kassambara et al., 2019) in R (script NI10). Log rank p values are reported across four expression quartiles. Cox hazard regression model was computed for comparison of quartile 1 (low expressors) versus quartile 4 (high expressors).

#### Analysis of RT PCR assay

Fold Change was calculated by determining the ratio of mRNA levels to control (day 2) values using the delta threshold cycle (Ct) method (DCt). A t test was used to find the significance of change between baseline (day 2) and treated time points (days 7, 19 and 70) based on normalized Cts to baseline (script NI14). Plots were made using ‘ggplot2′ (Wickham, 2016) in R.

#### Analysis of Wnt/β-catenin inhibition

Analysis was performed using CellInsight (Thermo Fisher Scientific) companion software across technical quadruplicates. The DMSO treated condition and single agent Wnt/β-catenin inhibitors reached confluency after 3 days in culture (100% maximum cut-off value). Significance values were calculated using a Student’s t test calculated at treatment endpoints (day 6).

### Additional Resources

Detailed protocols for single cell dissociation of small tumor biopsies (https://doi.org/10.17504/protocols.io.65rhg56) and high throughput smartseq2 libraries (https://doi.org/10.17504/protocols.io.2uwgexe) are available at protocols.io.

## Supplemental Information

Table S1. Sample Metadata, Related to Figure 1 Excel file containing four sub-sheets that describe 1) Sample clinical demographics 2) Sample cell types 3) Cancer cell annotation and 4) Sample analyses.

Table S2. Gene Lists, Related to Figures 1, 2, and 3 Excel file containing four sheets of gene lists including 1) Cell type markers 2) COSMIC tier 1 genes 3) Genes included in each cancer cell signature and 4) Immune markers.

Table S3. Detailed Mutation Analysis, Related to Figure 2 Excel file containing four sheets of data with specifics of the scRNaseq mutation analysis including 1) Summary of mutations 2) Summary of clinical drivers 3) scRNaseq mutations by cluster and 4) scRNaseq mutations by cell.

Table S4. Detailed Differentially Expressed Genes, Related to Figures 3 and 5 Detailed summary of differentially expressed genes among all comparison groups.

Table S5. Highlighted Differentially Expressed Genes, Related to Figures 3 and 5 Highlighted differentially expressed genes in each comparison group.

Table S6. Survival Analysis Statistics, Related to Figure 3 TCGA survival analysis for cancer cell signatures and macrophage abundance.
